# Supplementary material for: A Rho GTPase-effector ensemble governs cell migration behavior
Source: Nat Commun. 2025 Oct 31;16:9637. doi: 10.1038/s41467-025-64635-0 (PMC12579213; doi:10.1038/s41467-025-64635-0)
Supplement: Supplementary file 1 — Supplementary Information [file 41467_2025_64635_MOESM1_ESM.docx]

|  | Constructs | Information |
| --- | --- | --- |
| Rho family | DsRed-Rac1 | Q61L, ΔPB and ΔCAAX |
|  | DsRed-Rac2 | Q61L, ΔCAAX |
|  | DsRed-Rac3 | Q61L, ΔCAAX |
|  | DsRed-RhoG | Q61L, ΔCAAX |
|  | DsRed-Cdc42h | Q61L, ΔCAAX |
|  | DsRed-TC10 | Q75L, ΔCAAX |
|  | DsRed-TCL | Q79L, ΔCAAX |
|  | DsRed-RhoA | Q63L, ΔCAAX |
|  | DsRed-RhoB | Q63L, ΔCAAX |
|  | DsRed-RhoC | Q63L, ΔCAAX |
|  | DsRed-RhoD | Q75L, ΔCAAX |
|  | DsRed-RhoH | S13V, ΔCAAX |
|  | DsRed-Rho6 | S71L, ΔCAAX |
|  | DsRed-Rho7 | A16V, ΔCAAX |
|  | DsRed-RhoF | Q77L, ΔCAAX |
|  | DsRed-Rac1F37A | F37A, Q61L, ΔPB and ΔCAAX |
|  | DsRed-Rac1F37W | F37W, Q61L, ΔPB and ΔCAAX |
| Effector domains | CRIB_PAK1_-CFP-FT | 69-108 |
|  | CRIB_PAK2_-CFP-FT | 69-94 |
|  | CRIB_PAK3_-CFP-FT | 65-89 |
|  | CRIB_PAK4_-CFP-FT | 10-55 |
|  | CRIB_PAK6_-CFP-FT | 11-55 |
|  | CRIB_PAK7_-CFP-FT | 11-55 |
|  | CRIB_WASP_-CFP-FT | 230-287 |
|  | CRIBN_WASP_-CFP-FT | 203-239 |
|  | RBD_MRCKα_-CFP-FT | 1558-1593 |
|  | RBD_MRCKβ_-CFP-FT | 1583-1619 |
|  | RBD_ROCK1_-CFP-FT | 934-1015 |
|  | RBD_ROCK2_-CFP-FT | 964-1045 |
|  | RBD_DIAPH1_-CFP-FT | 63-260 |
|  | RBD_DIAPH2_-CFP-FT | 88-272 |
|  | RBD_DIAPH3_-CFP-FT | 118-287 |
|  | RBD_FMNL2_-CFP-FT | 23-276 |
|  | RBD_FMNL3_-CFP-FT | 27-278 |
|  | RBD_DAAM1_-CFP-FT | 45-233 |
|  | RBD_DAAM2_-CFP-FT | 41-229 |

Supplementary Table. 1. A list of DNA constructs used for INSPECT analysis of interactions between Rho small GTPases and effectors


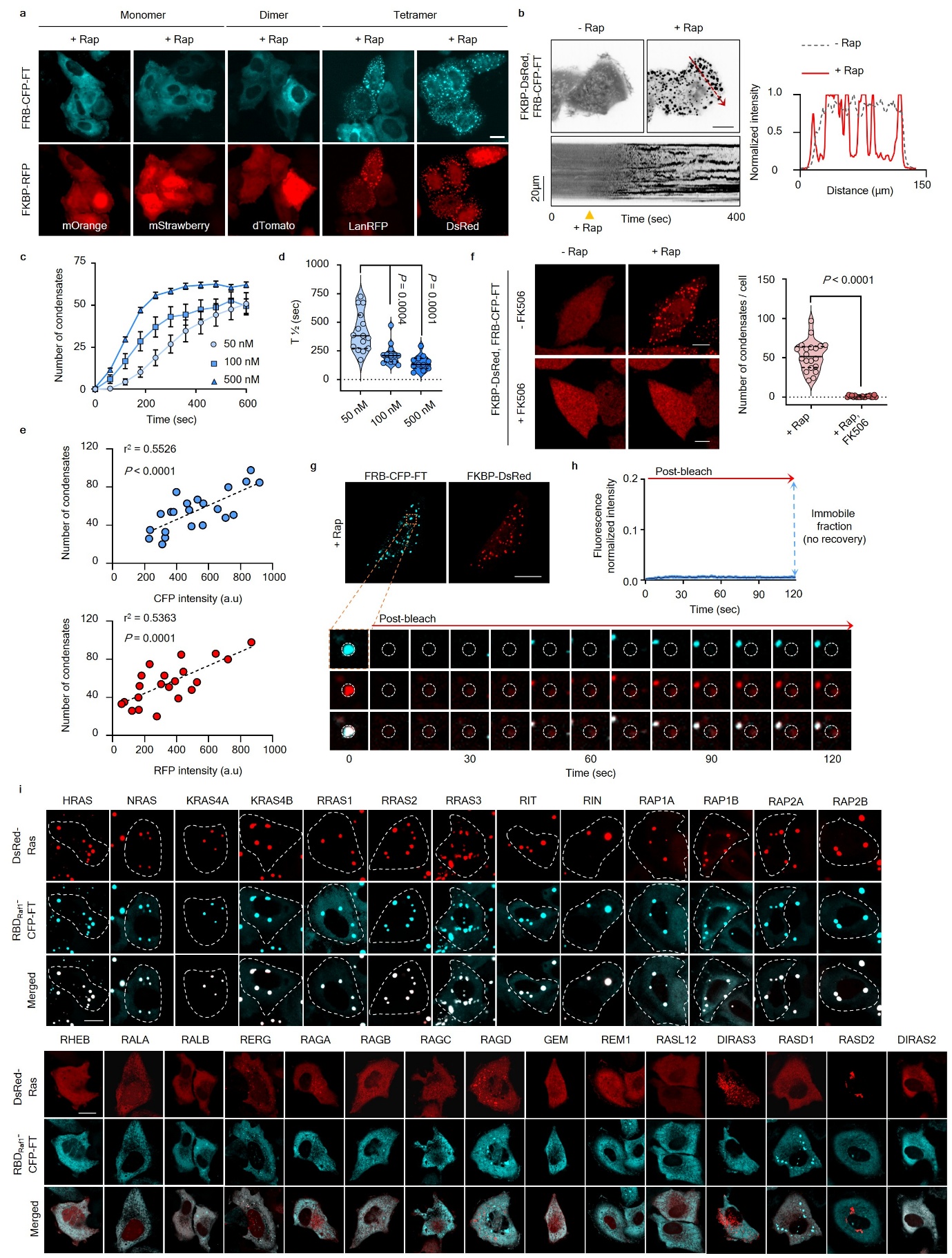


**Supplementary Fig. 1. Development of INSPECT to detect and visualize protein-protein interactions using phase-separated condensates in living cells.** (**a**) HeLa cells expressing FRB-CFP-FT (cyan) and FKBP-RFP (Red) are shown. After 500 nM rapamycin treatment, condensate formation was observed only in tetramers (LanRFP and DsRed) but not in monomers (mOrange, mStrawberry) or dimer (dTomato). (**b**) Representative images of HeLa cells expressing FRB-CFP-FT and FKBP-DsRed before and after treatment with 500 nM rapamycin. Kymograph of FKBP-DsRed (inverted) from the red dotted line over time. The yellow arrow indicates rapamycin addition. Line profiles of fluorescence intensity (RFP) correspond to the red dotted line indicated in the image. (**c**) Number of condensates was measured in the presence of 50, 100, and 500 nM rapamycin by time course (n = 15, 13 and 11 cells, N = 3). Graph showing rapamycin concentration-dependent condensate formation. Means and SEM are shown. (**d**) Violin plot shows the half-time (t_1/2_) of maximal condensates at each concentration (n ≥ 11 cells, N = 3). Median value, upper and lower quartiles (25th and 75th percentiles, dotted line) are shown. (**e**) Correlation between the number of condensates and fluorescence intensity in HeLa cells co-expressing FRB-CFP-FT and FKBP-DsRed. The linear regression fit was shown as a black dotted line, and the regression coefficient (r^2^) was shown. (**f**) HeLa cells co-transfected with FRB-CFP-FT and FKBP-DsRed were tested by 500 nM rapamycin with or without FK506. Graph showing inhibition of rapamycin-induced condensate formation by pre-incubation with 25 μM FK506 (n = 20 cells each, N = 3). Median value, upper and lower quartiles (25th and 75th percentiles, dotted line) are shown. (**g**) Representative images of rapamycin-induced synthetic condensates in HeLa cells co-expressing FKBP-DsRed and FRB-CFP-FT. Time series images of bleached condensate in yellow dotted box. (**h**) Fluorescence recovery after photobleaching (FRAP) analysis of rapamycin-induced synthetic condensates of FKBP-DsRed and FRB-CFP-FT in HeLa cells. Graph showing fluorescence recovery curves of rapamycin-induced synthetic condensates (n = 16, N = 3). The same-sized area was bleached in multiple cells to measure CFP recoveries. Means and SEM are shown. (**i**) HeLa cells co-transfected with RBD_Raf1_-CFP-FT, DsRed-Ras are shown. The 13 Ras GTPases (HRas, NRas, KRas4A, KRas4B, RRas1, RRas2, RRas3, RIT, RIN, Rap1A, Rap1B, Rap2A, Rap2B) can directly interact with RBD_Raf1_. Representative images shown are from ≥ 3 independent experiments with similar results. Scale bars, 20 μm (all panels). P-value were determined by the one-way ANOVA with Dunnett’s post hoc test for multiple comparisons in (d). Unpaired two-tailed t-test P-value is shown in (f).


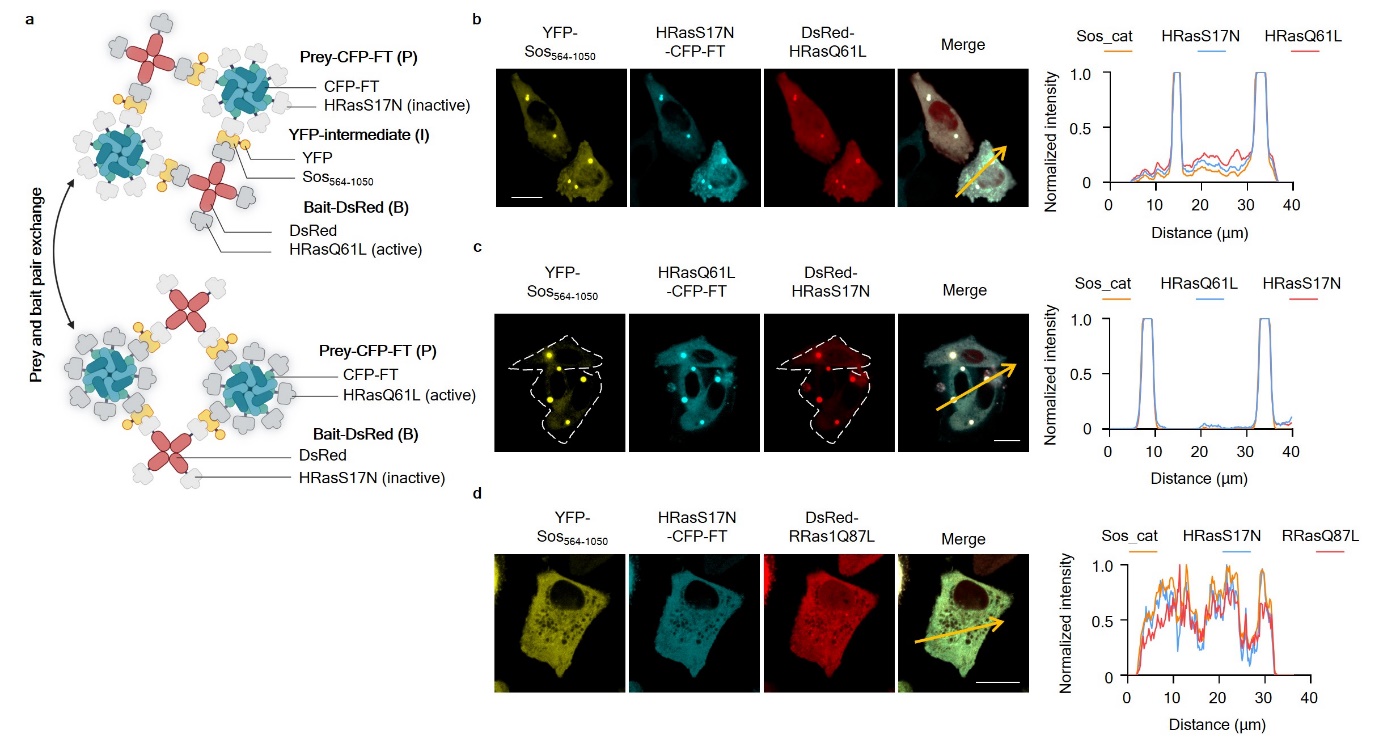


**Supplementary Fig. 2. INSPECT detects the ternary protein interactions in living cells.** (**a**) Scheme of Phase-separated condensates for detecting ternary protein complexes by co-expression of fluorescent protein conjugated-intermediate protein (I) with prey-CFP-FT (P) and DsRed-bait (B). Schematic was created in BioRender. Heo, W. (2025) https://BioRender.com/2718swf. (**b**) HeLa cells co-transfected with HRasS17N-CFP-FT (inactive), DsRed-HRasQ61L (active), and YFP-Sos_564-1050_ (catalytic domain) are shown. Line profiles of fluorescence intensity corresponding to the yellow line indicated in the merged image. (**c**) HeLa cells are co-transfected with HRasQ61L (active)-CFP-FT, DsRed-HRasS17N (inactive), and YFP-Sos_564-1050_. Line scan profiles of fluorescence intensity corresponding to the yellow line indicated in merged images. Condensates were detected despite exchanging FT and DsRed pairs. (**d**) HeLa cells are co-transfected with HRasS17N (inactive)-CFP-FT, DsRed-RRasQ87L (active), and YFP-Sos_564-1050_. Line scan profiles of fluorescence intensity corresponding to the yellow line indicated in merged images. The active form of RRas1 (DsRed-RRas1Q87L) was used as a negative control, and no condensate formation was observed. Representative images shown are from ≥ 3 independent experiments with similar results. Scale bars, 20 μm.


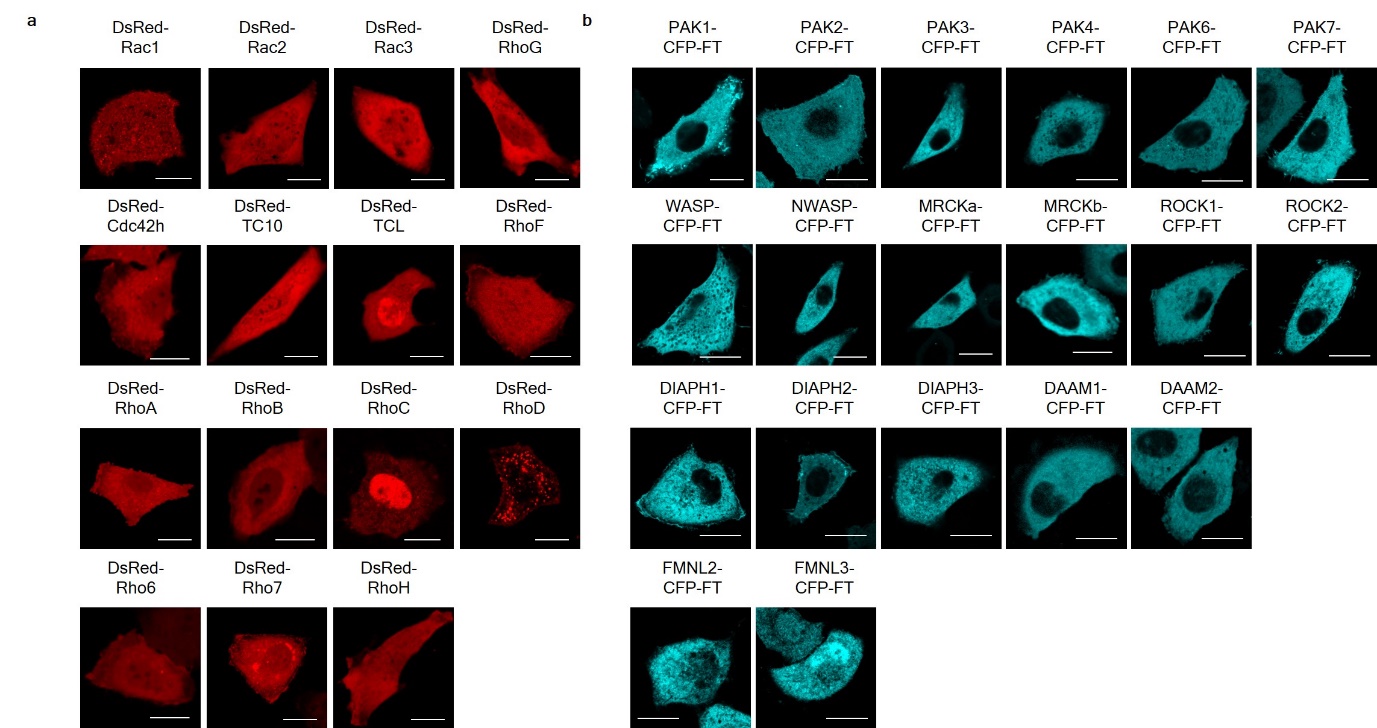


**Supplementary Fig. 3. DsRed-conjugated Rho GTPases and ferritin-conjugated effector proteins expression results.** (**a, b**) HeLa cells expressing DsRed-conjugated Rho GTPases and ferritin-conjugated effector proteins are shown. The CAAX motif was deleted from Rho GTPases to visualize Rho GTPase in the cytosol. However, some Rho GTPases showed nuclear localization (TCL, RhoC), possibly due to the remaining polybasic property. In the case of RhoD, it is sufficient to induce self-assembled condensates due to its homo-oligomerization properties. Representative images shown are from ≥ 3 independent experiments with similar results. Scale bars, 20 μm.


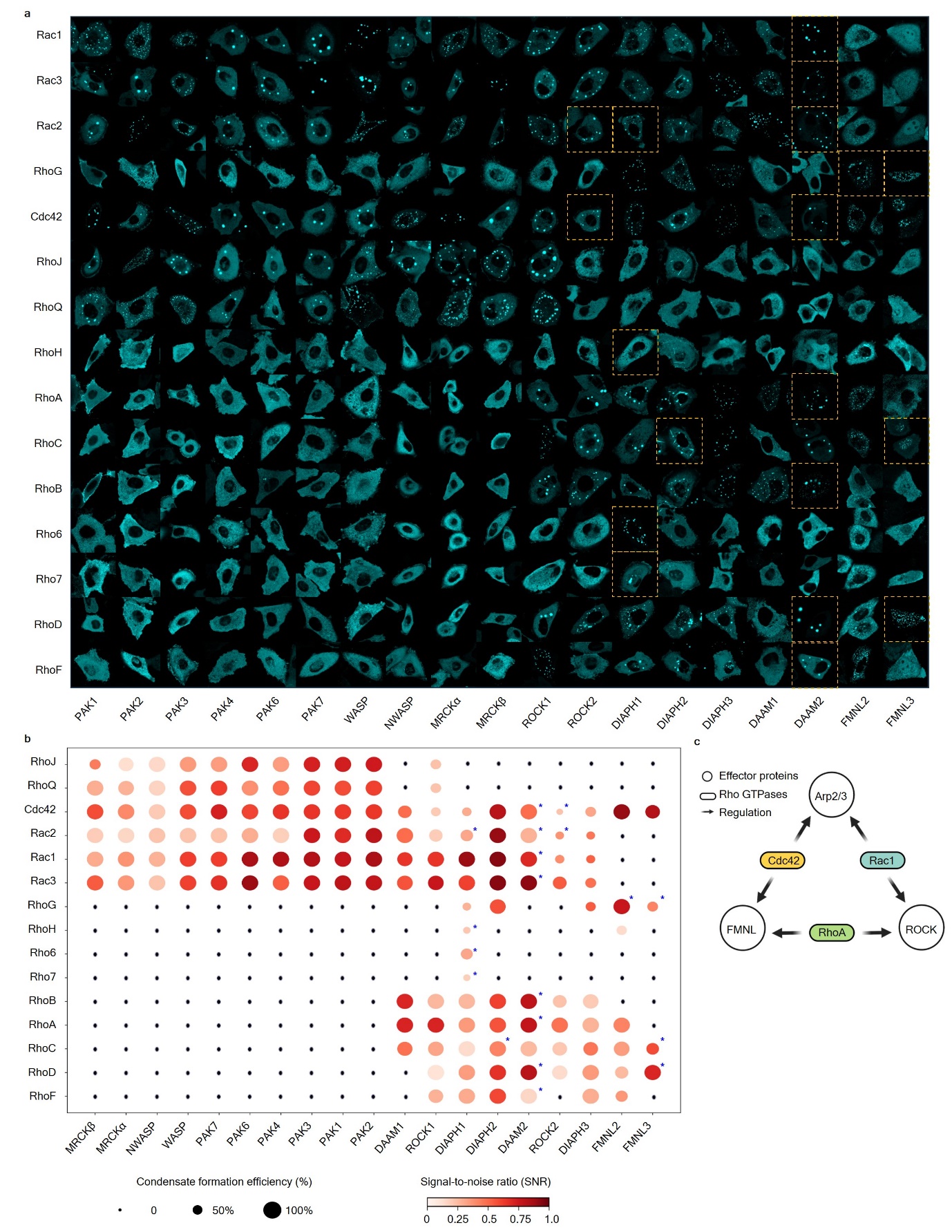


**Supplementary Fig. 4. INSPECT screening results for Rho GTPases and effector protein interactions.** (**a**) Representative images of Rho GTPases and effector interaction-induced condensates are shown, with newly identified 19 interaction pairs highlighted by yellow dotted boxes. In the case of the ferritin-conjugated effector-only expression, self-assembled condensates were not observed. Therefore, only images of the ferritin-conjugated effector are shown. (**b**) Molecular interaction map between Rho GTPases and their downstream effectors. Out of 285 pairs examined, 139 positive interactions were identified, and blue asterisks (*) indicate 19 newly identified interactions based on our screening platform. Condensate formation efficiency ranges from 0 to 100% and is depicted by the size of the circles. When efficiency is 0, it is shown as a black dot for better visibility. The signal-to-noise ratio (SNR) ranges from 0-1 and is represented by a one-color gradient within each circle (N > 5). (**c**) Simplified summary model of Rho GTPase and effector ensemble. Representative images shown are from ≥ 3 independent experiments with similar results. Schematic was created in BioRender. Heo, W. (2025) https://BioRender.com/2718swf.


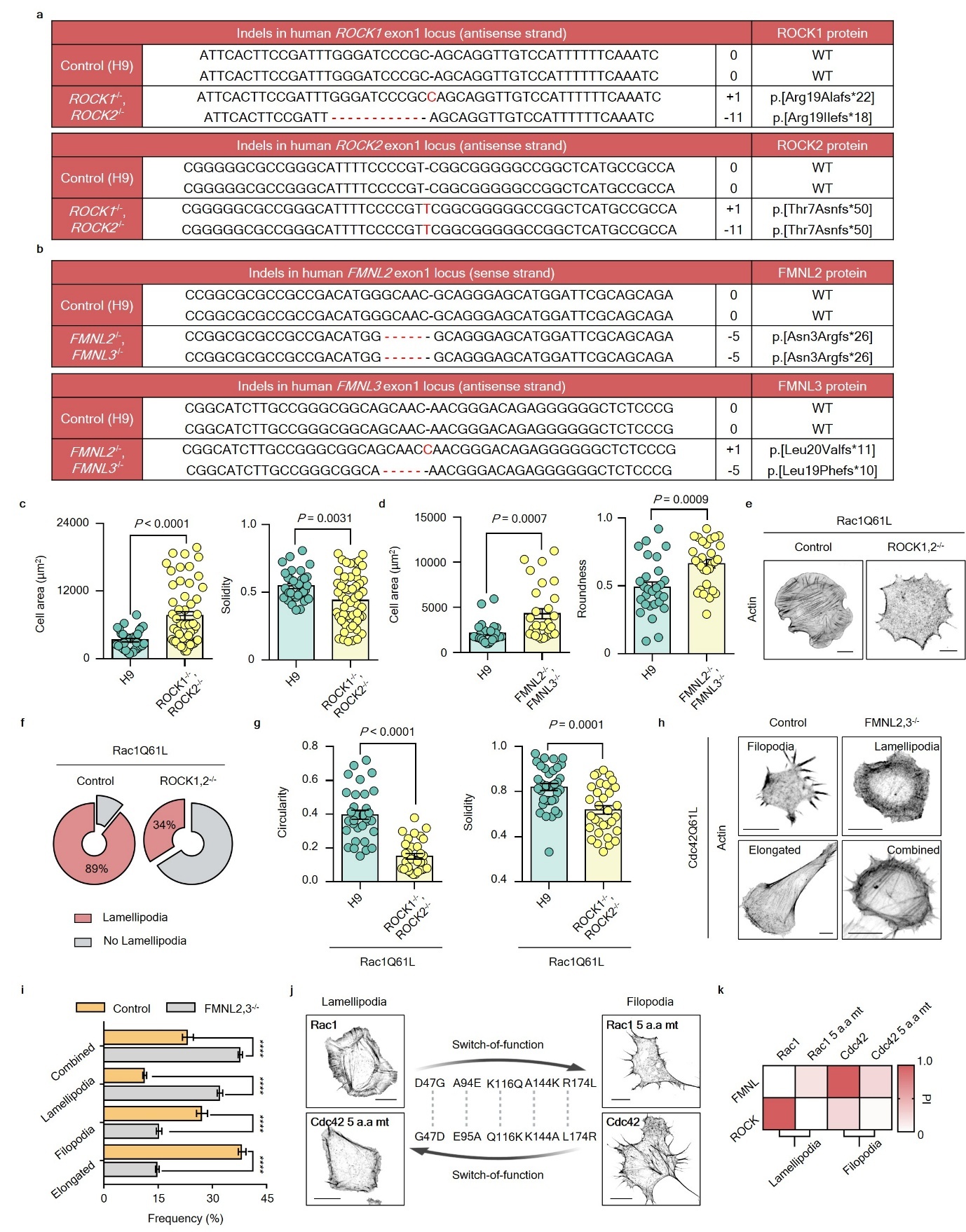


**Supplementary Fig. 5. FMNL and ROCK as key effectors transforming Cdc42 and Rac1 activities into distinct cellular morphology.** (**a, b**) Sequences depict both the wild type (WT) and the bi-allelic nonsense mutations caused by CRISPR/Cas9 in the targeted regions of the ROCK1 and ROCK2 genes (a), the FMNL2 and FMNL3 genes (b). These mutations arise either from a single nucleotide insertion (+1) or deletion (-1) or from deletions of five or eleven nucleotides (-5 or -11). Such changes lead to a frameshift, introducing a premature stop codon and, thus, gene knockout. The hESC lines with double knockouts (ROCK1^-/-^, ROCK2^-/-^ or FMNL2^-/-^, FMNL3^-/-^) were subsequently expanded and differentiated for desired experiments. (**c**) Quantification of cell area and solidity in H9 and ROCK1,2^-/-^ (n= 35 and 54 cells, N= 3) cells expressing mCh-LifeAct. Means and SEM are shown. (**d**) Quantification of cell area and roundness in H9 and FMNL2,3^-/-^ (n= 28 and 29 cells, N= 3) cells expressing mCh-LifeAct. Means and SEM are shown. (**e**, **f**) Representative images and morphological analysis of ROCK1,2^-/-^ and H9 control cells expressing mCh-LifeAct with CFP-Rac1Q61L. (**g**) Quantification of cell circularity and solidity in H9 and ROCK1,2^-/-^ (n= 32 cells each, N= 3) cells expressing mCh-LifeAct with CFP-Rac1Q61L. Means and SEM are shown. (**h**, **i**) Representative images and morphological analysis of FMNL2,3^-/-^ and H9 control cells expressing mCh-LifeAct with CFP-Cdc42Q61L (control, n = 77 cells, FMNL2,3^-/-^, n = 120 cells, N= 3). Means and SEM are shown. Statistical significance was determined by two-way ANOVA with Sidak’s multiple comparison test. (**j**) Representative images lamellipodia and filopodia formation in NIH3T3 cells expressing CFP-Rac1 5 a.a mt and CFP-Cdc42 5 a.a mt with mCh-LifeAct. These mutants exchange the activities of Rac1 and Cdc42 to visualize changes in protrusion morphology. Schematic was created in BioRender. Heo, W. (2025) https://BioRender.com/2718swf. (**k**) Screening of interactions between Rac1, Rac1 5 a.a mt, Cdc42, Cdc42 5 a.a mt with FMNL and ROCK proteins. All representative images shown are from ≥ 3 independent experiments with similar results. Scale bars, 20 μm (all panels). Unpaired two-tailed t-test P-values are shown in (c), (d), and (g).


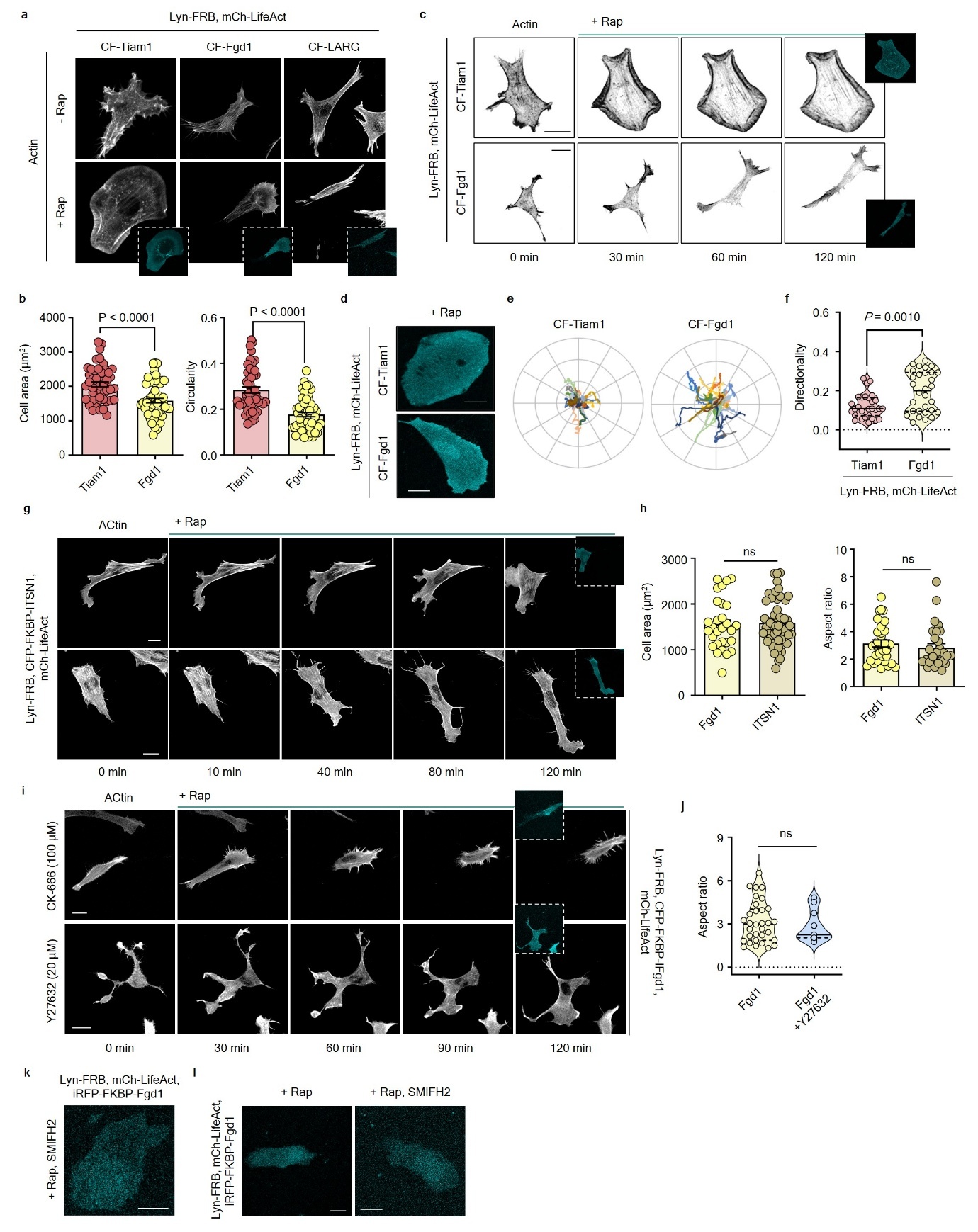


**Supplementary Fig. 6. Formin family proteins are required for Cdc42-induced symmetry breaking.** (**a**) Representative images of NIH 3T3 cells expressing Lyn-FRB, mCh-LifeAct, and CF-Tiam1, CF-Fgd1 or CF-LARG. These images show the corresponding morphological changes induced by each Rac1-, Cdc42-, and RhoA-specific GEF before and after treatment with 500 nM rapamycin. (**b**) Quantification of cell area and circularity in NIH 3T3 cells expressing Lyn-FRB, mCh-LifeAct, CF-Tiam1 or CF-Fgd1 (n= 49 and 57 cells, N=3). Cells were treated with 500 nM rapamycin. Means and SEM are shown. (**c**) Global activation of Rac1-GEF (Tiam1) or Cdc42-GEF (Fgd1) caused distinct morphological changes. Even with global activation, Cdc42-activated cells showed polarized morphology. (**d**) Representative images of CF-Tiam1 (upper) and CF-Fgd1 (lower) corresponding to mCh-LifeAct images shown in Fig. 2f. Scale bars, 20 μm. (**e, f**) Cell trajectories were plotted from the origin in Rac1- or Cdc42-activated cells. Graph showing directionality in NIH 3T3 cells expressing Lyn-FRB and CF-Tiam1 or CF-Fgd1 (n= 30 cells each, N=3). Cells were treated with 500 nM rapamycin. The median value, upper and lower quartiles (25th and 75th percentiles, dotted line) are shown. (**g**) Representative images of NIH 3T3 cells expressing Lyn-FRB, mCh-LifeAct, and CF-ITSN1. Cells showed polarized morphology induced by activated Cdc42 when treated with 500 nM rapamycin. (**h**) Quantification of cell area and aspect ratio in NIH 3T3 cells expressing Lyn-FRB, mCh-LifeAct, and CF-Fgd1 or CF-ITSN1 (n≥ 32 and 29 cells, N=3). Cells were treated with 500 nM rapamycin. Means and SEM are shown. (**i**) Representative images of NIH 3T3 cells expressing Lyn-FRB, mCh-LifeAct, and CF-Fgd1. Cells were treated with 100 μM CK-666 (upper) or 20 μM Y27632 (lower) for 2 hours and then treated with 500 nM rapamycin. (**j**) Quantification of aspect ratio in control and Y27632-treated cells (n= 11 cells, N=3). Median value, upper and lower quartiles (25th and 75th percentiles, dotted line) are shown. (**k**) Representative image of CF-Fgd1 corresponding to the cell shown in Figure 2h. (**l**) Representative images of CF-Fgd1 corresponding to the cells shown in Figure 2i. The left image was captured before rapamycin treatment. All representative images shown are from ≥ 3 independent experiments with similar results. Scale bars, 20 μm (all panels). Unpaired two-tailed t-test P-values are shown in (b), (f), (h) and (j).


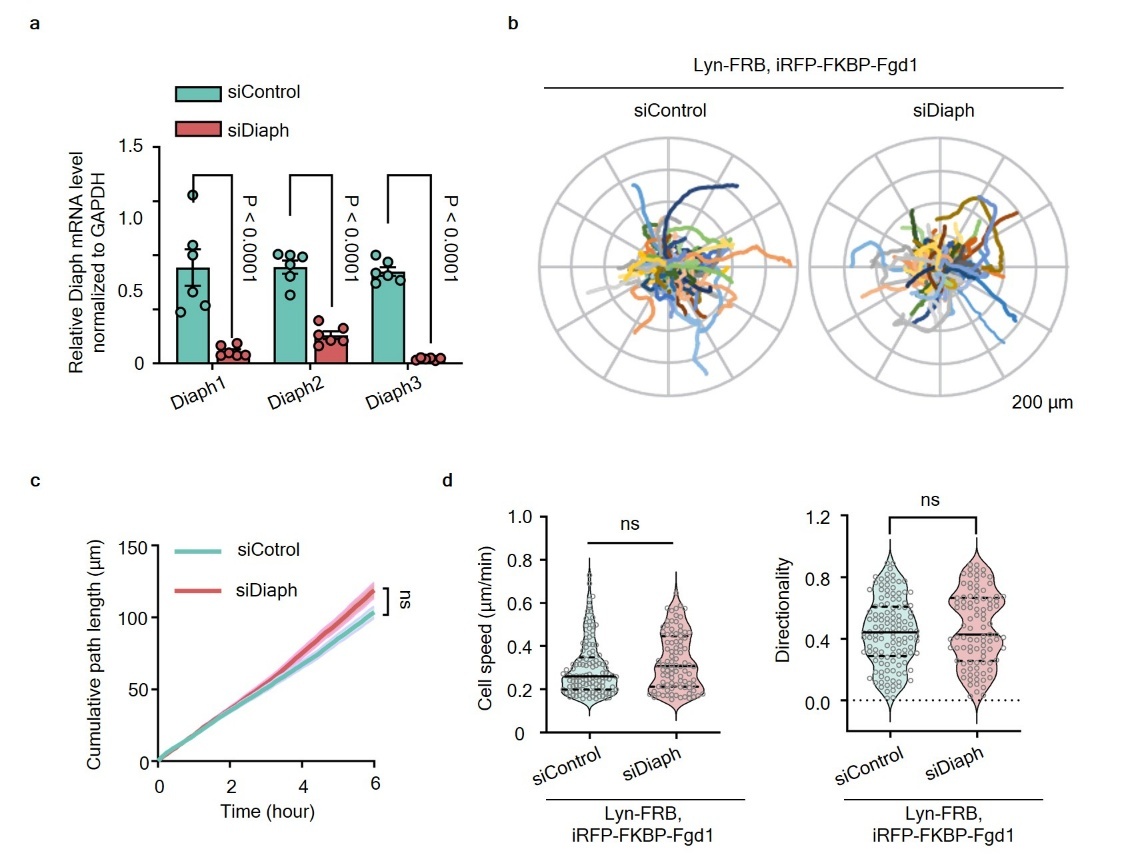


**Supplementary Fig. 7. DIAPHs are dispensable for Cdc42 activation-induced polarity establishment.** (**a**) The relative mRNA levels were analyzed in control and Diaph1, 2, and 3-depleted cells by qRT-PCR (N= 6 each). Means and SEM are shown. Statistical significance was determined by two-way ANOVA with Sidak’s multiple comparison test. (**b**) Cell trajectories were plotted from the origin in control and Diaph1/2/3-depleted NIH3T3 cells expressing Lyn-FRB, iRFP-FKBP-Fgd1, and mCh-LifeAct. Cells were treated with rapamycin and monitored for 6 hours. (**c**, **d**) Analysis of cumulative path length, speed, and directionality in control (n = 126 cells) and Diaph1/2/3-depleted (n = 95 cells) NIH3T3 cells expressing Lyn-FRB, iRFP-FKBP-Fgd1, and mCh-LifeAct. Median value, upper and lower quartiles (25th and 75th percentiles, dotted line) are shown. Statistical significance in (d) was determined by unpaired two-tailed t-test.


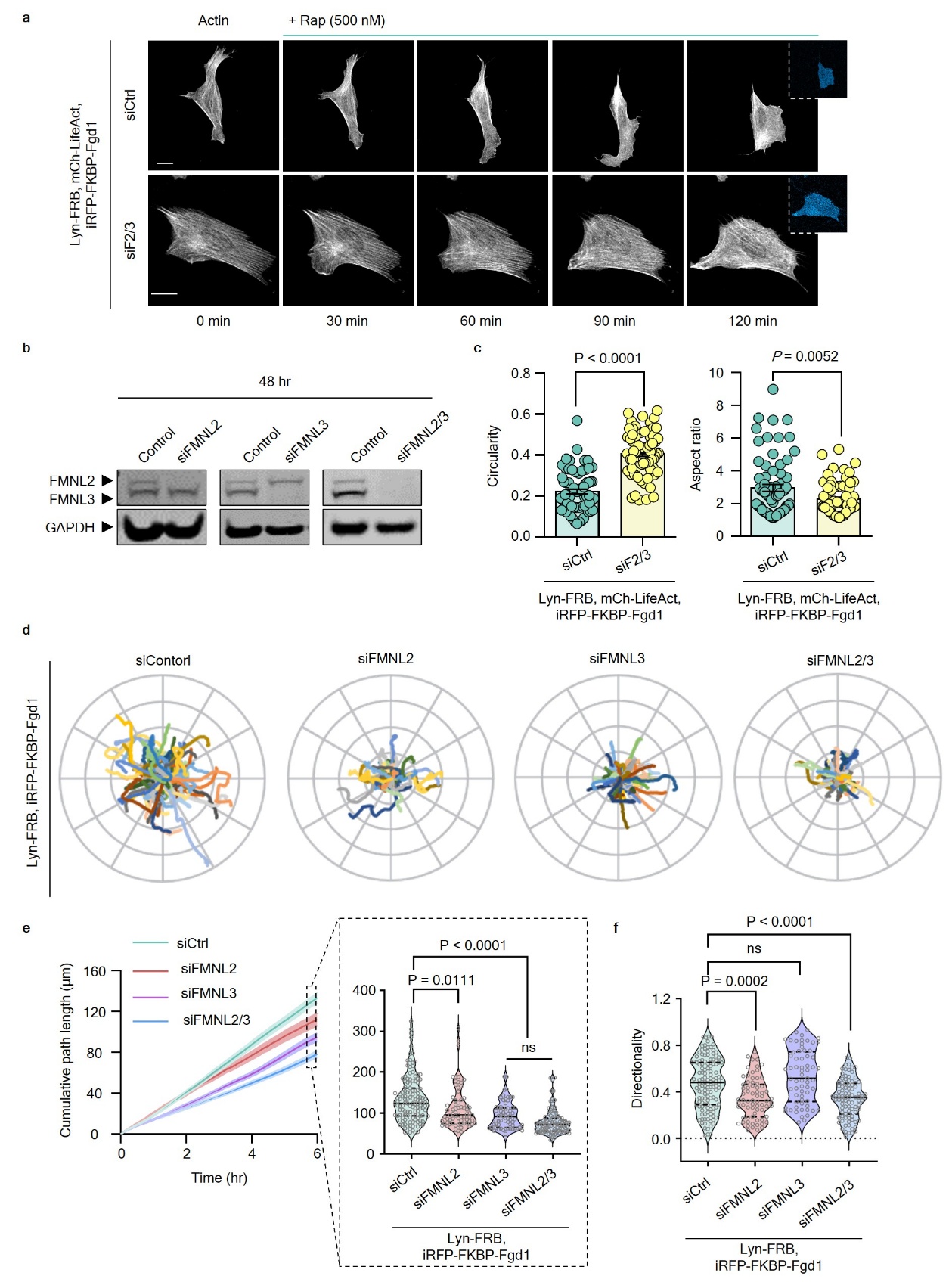


**Supplementary Fig. 8. Depletion of FMNL2 and FMNL3 affects cell migration behavior induced by Cdc42 activation.** (**a**) Representative images of control and FMNL2/3-depleted NIH3T3 cells expressing Lyn-FRB, iRFP-FKBP-Fgd1, and mCh-LifeAct. Cells were treated with rapamycin and monitored for 2 hours. Representative images shown are from ≥ 3 independent experiments with similar results. Scale bars, 20 μm. (**b**) NIH3T3 cells were transfected with siRNA against control, FMNL2, FMNL3, and FMNL2/3. After 48 hours of transfection, the expression changes of FMNL2 and FMNL3 were detected by western blot (N=3). The GAPDH was used as an internal control. (**c**) Quantification of cell circularity and aspect ratio in control and FMNL2/3-depleted NIH3T3 cells expressing Lyn-FRB, mCh-LifeAct, and iRFP-FKBP-Fgd1 (n= 72 and 75 cells, N=3). Means and SEM are shown. (**d**) Cell trajectories were plotted from the origin in control and FMNL2, FMNL3, and FMNL2/3-depleted NIH3T3 cells expressing Lyn-FRB and iRFP-FKBP-Fgd1. Cells were treated with rapamycin and monitored for 6 hours. (**e**, **f**) Analysis of cumulative path length and directionality in control (n= 131 cells) and FMNL2 (n = 70 cells), FMNL3 (n = 66 cells), and FMNL2/3-depleted (n= 92 cells) NIH3T3 cells expressing Lyn-FRB and iRFP-FKBP-Fgd1. Cells were treated with rapamycin and monitored for 6 hours. Violin plot in panel (e) shows the cumulative path length at the last time point (6 hours), and the violin plot in panel (f) shows directionality. Median value, upper and lower quartiles (25th and 75th percentiles, dotted line) are shown). Unpaired two-tailed t-test P-values are shown in (c). *P*-values were determined by the one-way ANOVA with Turkey’s post hoc test in (e) and (f).

**
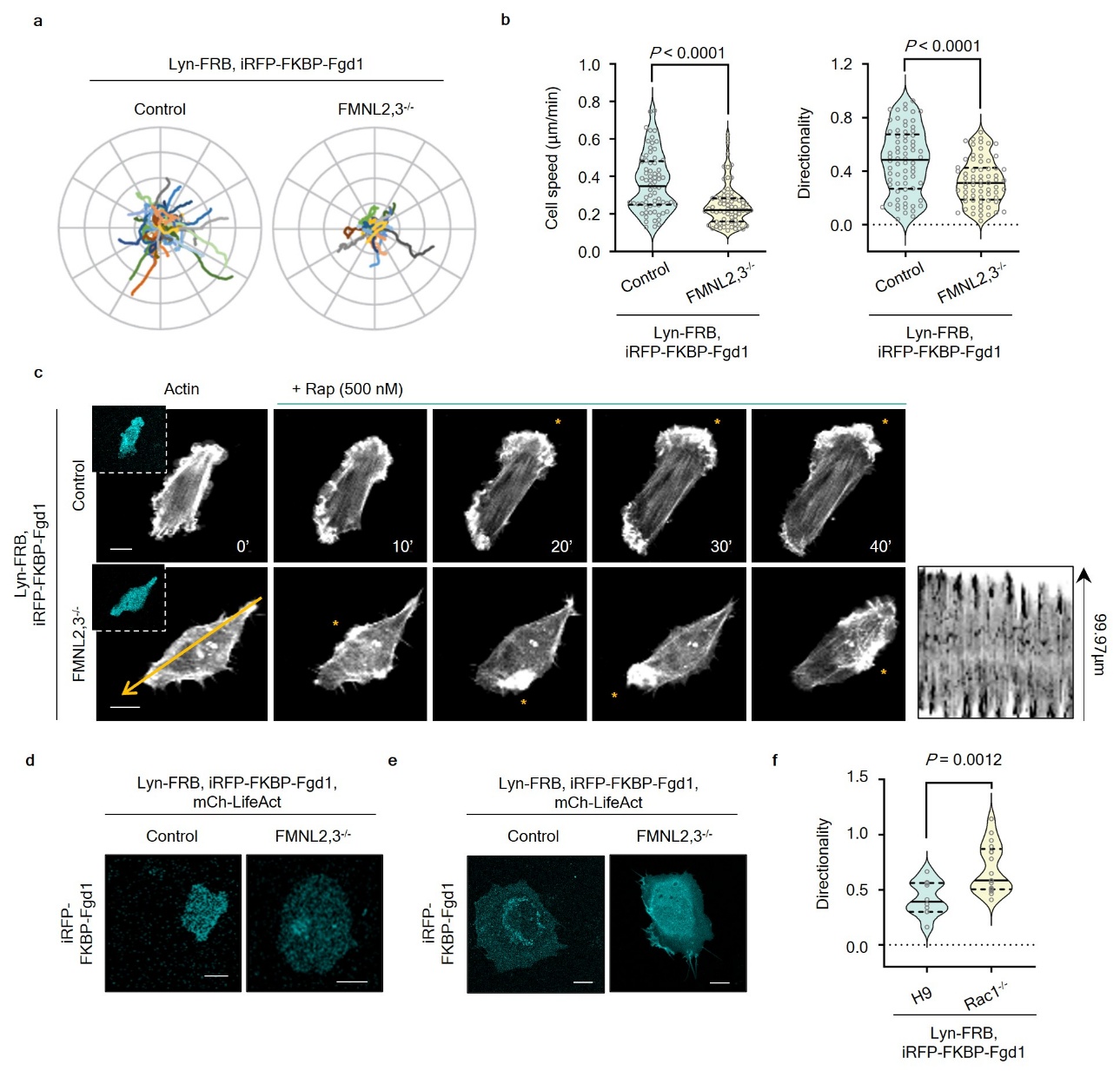
**

**Supplementary Fig. 9. FMNL2 and FMNL3 are required for Cdc42-induced symmetry breaking through actin enrichment.** (**a**) Cell trajectories were plotted from the origin in control (H9) and FMNL2/3-knockout cells expressing Lyn-FRB and iRFP-FKBP–Fgd1. Cells were treated with rapamycin and monitored for 6 hours. (**b**) Analysis of speed and directionality in control (H9) and FMNL2,3^-/-^ cells expressing Lyn-FRB, mCh-LifeAct, and iRFP-FKBP-Fgd1 (n= 70 and 68 cells, N= 3). Cells were treated with rapamycin and monitored for 6 hours. Median value, upper and lower quartiles (25th and 75th percentiles, dotted line) are shown. (**c**) Representative images of control (H9) and FMNL2,3^-/-^ cells expressing Lyn-FRB, mCh-LifeAct, and iRFP-FKBP-Fgd1. Cells were treated with rapamycin and monitored for 2 hours. The location of actin accumulation was monitored, and yellow asterisks indicate the region of actin enrichment. (d) Representative images of iRFP-FKBP-Fgd1 corresponding to mCh-LifeAct images shown in Fig. 2j. The images were captured 3 hours after treatment with 500 nM rapamycin. (e) Representative images of iRFP-FKBP-Fgd1 corresponding to mCh-LifeAct images shown in Fig. 2k. The images were captured 1 hour after treatment with 500 nM rapamycin. (**f**) Violin plot shows directionality in control and Rac1^-/-^ (n = 21 and 50 cells, N=3) expressing Lyn-FRB and iRFP-FKBP-Fgd1. Cells were treated with 500 nM rapamycin. Median value, upper and lower quartiles (25th and 75th percentiles, dotted line) are shown. Unpaired two-tailed t-test P-values are shown in (c) and (f). Representative images shown are from ≥ 3 independent experiments with similar results. Scale bars, 20 μm (all panels).


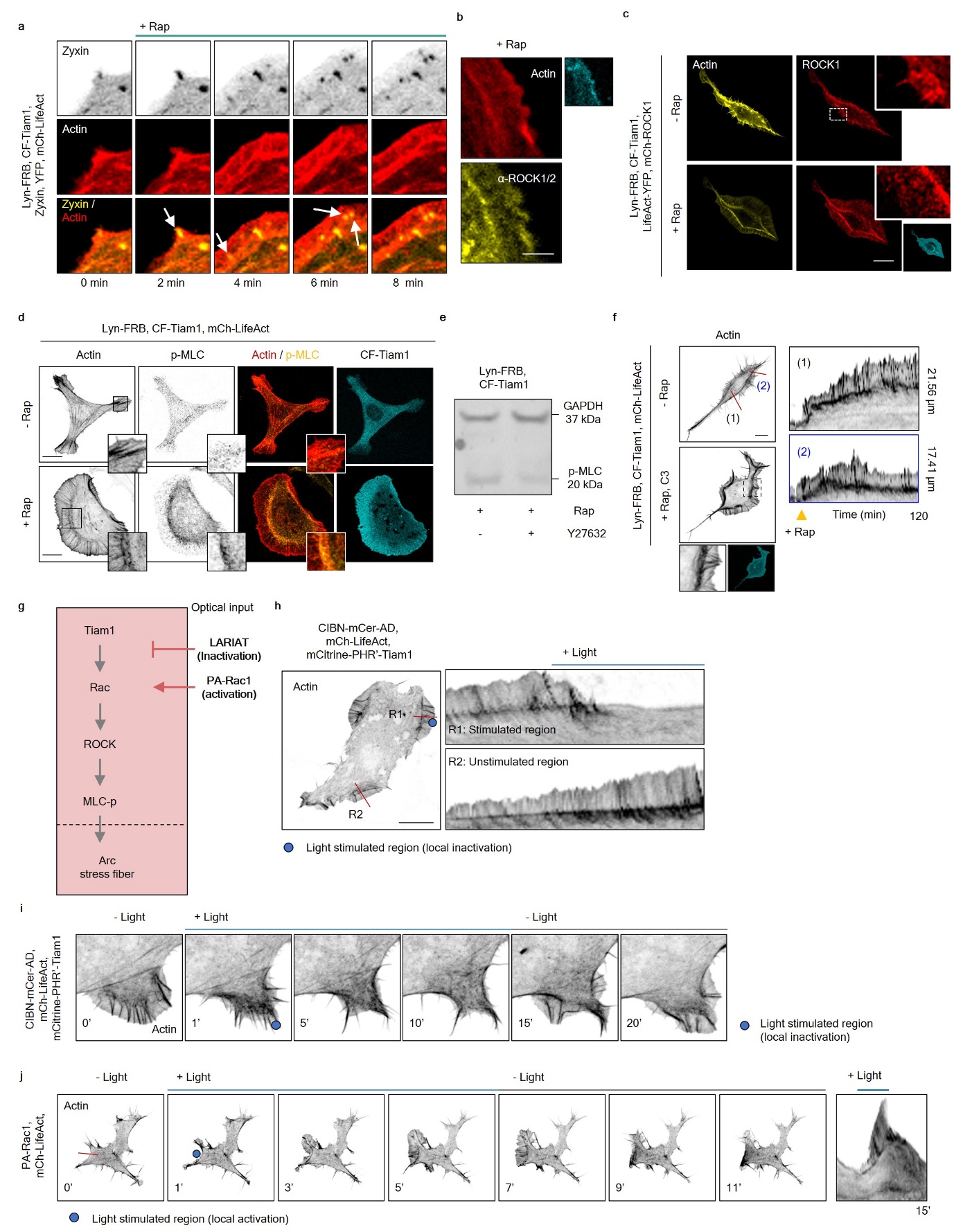


**Supplementary Fig. 10. Arc stress fiber generation via Rac/ROCK/MLC is recapitulated through optogenetic modulation of Rac activity.** (**a**) Time-lapse montages of mCh-LifeAct (actin) and Zyxin-YFP (zyxin) showing newly forming arc stress fiber and adhesion after rapamycin treatment. (**b**) Fluorescence images showing endogenous ROCK1/2 localization after Rac1 activation in NIH3T3 cells. Scale bar, 5 μm. (**c**) Exogenous ROCK1 localization after Rac1 activation in NIH3T3 cells expressing Lyn-FRB, CF-Tiam1, LifeAct-YFP, and mCh-ROCK1. (**d**) NIH3T3 cells expressing Lyn-FRB, CF-Tiam1 with or without rapamycin treatment. The p-MLC (Ser-19; yellow) co-localizes with the F-actin bundle (arc stress fiber). (**e**) Western blot shows p-MLC (Ser-19) levels change upon Y27632 treatment. Lysates were immunoblotted for p-MLC and GAPDH (loading control). (**f**) Cells were pre-incubated with C3 exoenzyme (0.2 μgml^-1^) to inhibit RhoA activation. After 2 hours, cells were treated with 500 nM rapamycin. The RhoA-inhibited cell showed the failure of arc SFs retrograde flow toward the cell center. (**g**) Schematic representation of optogenetic modulation of arc SFs generation and disruption. Different optogenetic tools were used to activate (PA-Rac1) or deactivate (LARIAT) Rac. (**h, i**) NIH3T3 cells expressing mCitrine-PHR’-Tiam1, CIBN-mCer-AD, and mCh-LifeAct. Local inactivation (blue circle) of Tiam1 showed membrane retraction in the stimulated region (R1), while the unstimulated region (R2) showed enforced protrusion and arc SFs. (**j**) NIH3T3 cells expressing PA-Rac1 and mCh-LifeAct. The local activation (blue circle) of PA-Rac1 showed membrane protrusion in the stimulated area and robust LP formation with arc SFs. Representative images shown are from ≥ 3 independent experiments with similar results. Scale bars, 20 μm

**
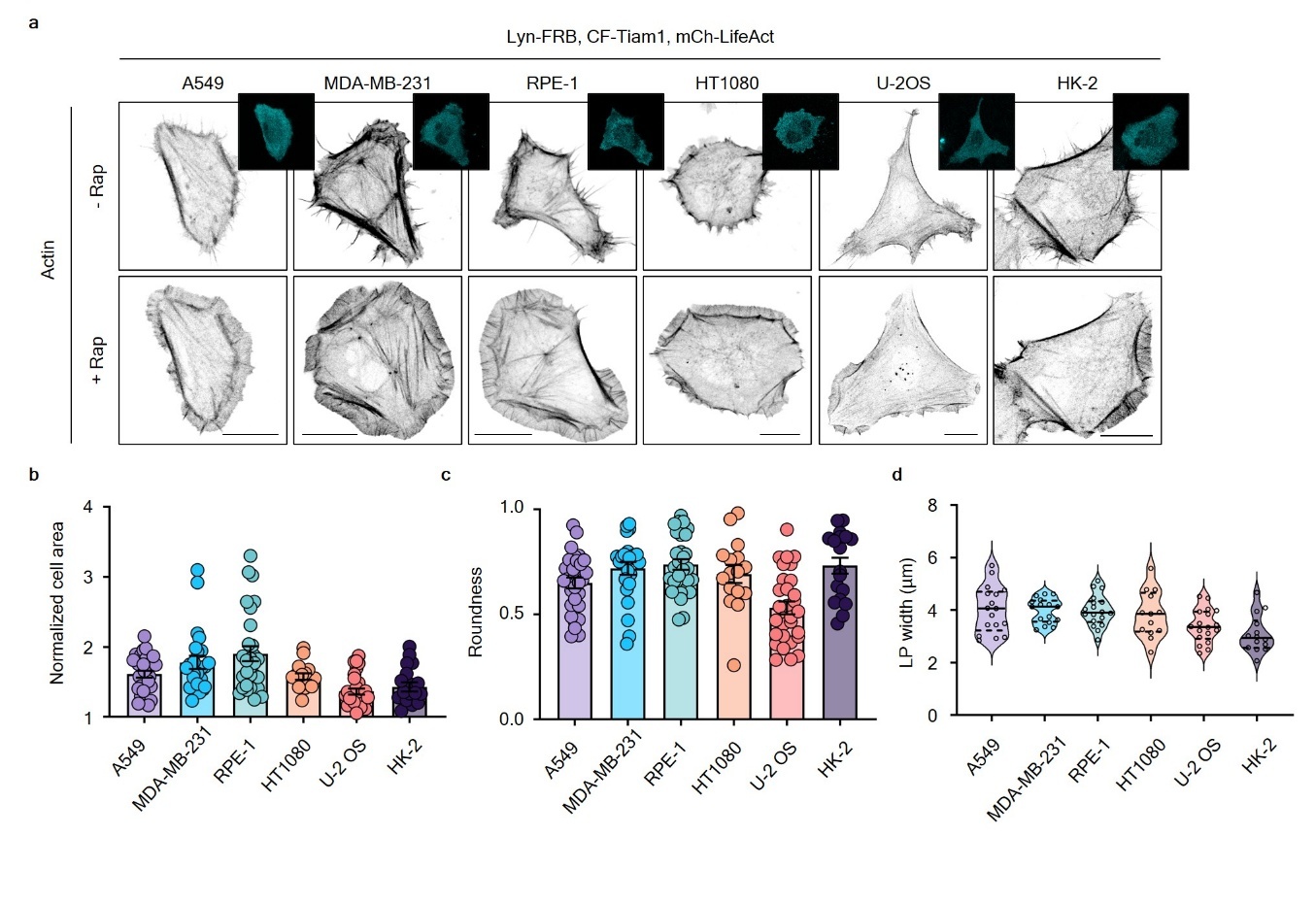
Supplementary Fig. 11. Rac/ROCK-induced arc stress fiber formation observed across various cell types.** (**a**) Representative images of A549, MDA-MB-231, RPE-1, HT1080, U-2OS and HK-2 cells expressing Lyn-FRB, mCh-LifeAct (inverted contrast), and CF-Tiam1. Cells were treated with 500 nM rapamycin. Representative images shown are from ≥ 3 independent experiments with similar results. Scale bars, 20 μm. (**b**, **c**) Quantification of normalized cell area and roundness in various cells expressing Lyn-FRB, mCh-LifeAct, CF-Tiam1. Cells were treated with 500 nM rapamycin. (n= 30, 24, 29, 16, 30 and 19 cells, N=3). Means and SEM are shown. (**d**) Graph showing lamellipodia width, the shortest vertical distance from the membrane to arc SFs in various cells expressing Lyn-FRB, mCh-LifeAct, CF-Tiam1 (n= 17, 16, 18, 13, 18 and 14 cells, N=3). Median value, upper and lower quartiles (25th and 75th percentiles, dotted line) are shown.

**
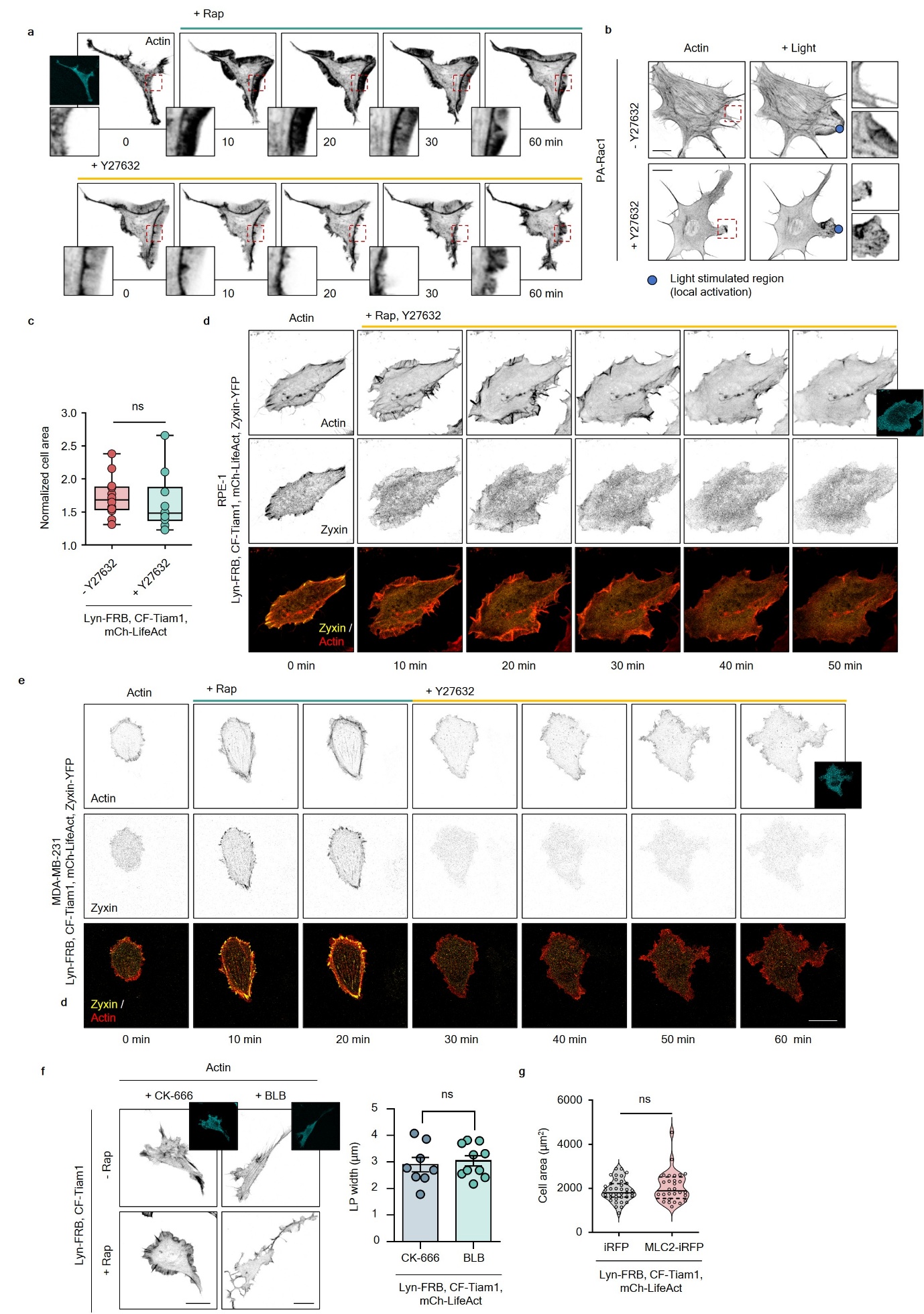
**

**Supplementary Fig. 12. Parallel activation of ROCK- and Arp2/3-mediated pathways converging in arc SF generation and regulation in lamellipodia protrusions.** (**a**) NIH 3T3 cells expressing Lyn-FRB, CF-Tiam1, mCh-LifeAct (inverted contrast). After 1 hour of rapamycin treatment (upper), cells were treated with 20 μM Y27632 (lower). (**b**) NIH3T3 cell expressing PA-Rac1 and mCh-LifeAct (inverted contrast). Cells were treated with or without 20 μM Y27632. Enlarged inset boxes show membrane protrusions after light stimulation. Cells treated with the ROCK inhibitor Y27632 failed to form arc SFs. (**c**) Graph showing normalized cell area in NIH 3T3 cells expressing Lyn-FRB, CF-Tiam1 and mCh-LifeAct. Cells were treated with 500nM rapamycin, with or without 20 μM Y27632 (n= 12 and 10 cells, N=3). Means and SEM are shown. (**d**) Time-lapse images of MDA-MB-231 cell expressing Lyn-FRB, CF-Tiam1, mCh-LifeAct (actin) and Zyxin-YFP (zyxin) showing newly forming arc stress fiber and adhesion after treatment with 500 nM rapamycin. Cells were treated with 20 μM of Y27632. (**e**) Time-lapse images of RPE-1 cell expressing Lyn-FRB, CF-Tiam1, mCh-LifeAct (actin) and Zyxin-YFP (zyxin). Cell treated with 500 nM rapamycin and 20 μM of Y27632. (**f**) NIH 3T3 cells expressing Lyn-FRB, CF-Tiam1 and mCh-lifeAct. Cells were pre-incubated with CK-666 or blebbistatin for 2 hours, and then cells were treated with 500 nM rapamycin (n= 8 and 10 cells, N=3). We measured the lamellipodia (LP) width, the shortest vertical distance from the PM to arc stress fibers. Means and SEM are shown. (**g**) Graph showing cell area in NIH3T3 cells expressing Lyn-FRB, CFP-FKBP-Tiam1, mCh-LifeAct, and iRFP682 or MLC2-iRFP682. Cells were treated with 500 nM rapamycin (n= 37and 28 cells, N=3). Median value, upper and lower quartiles (25th and 75th percentiles, dotted line) are shown. Representative images shown are from ≥ 3 independent experiments with similar results. Scale bars, 20 μm (all panels). Unpaired two-tailed t-test P-values are shown in (c), (f) and (g).

**
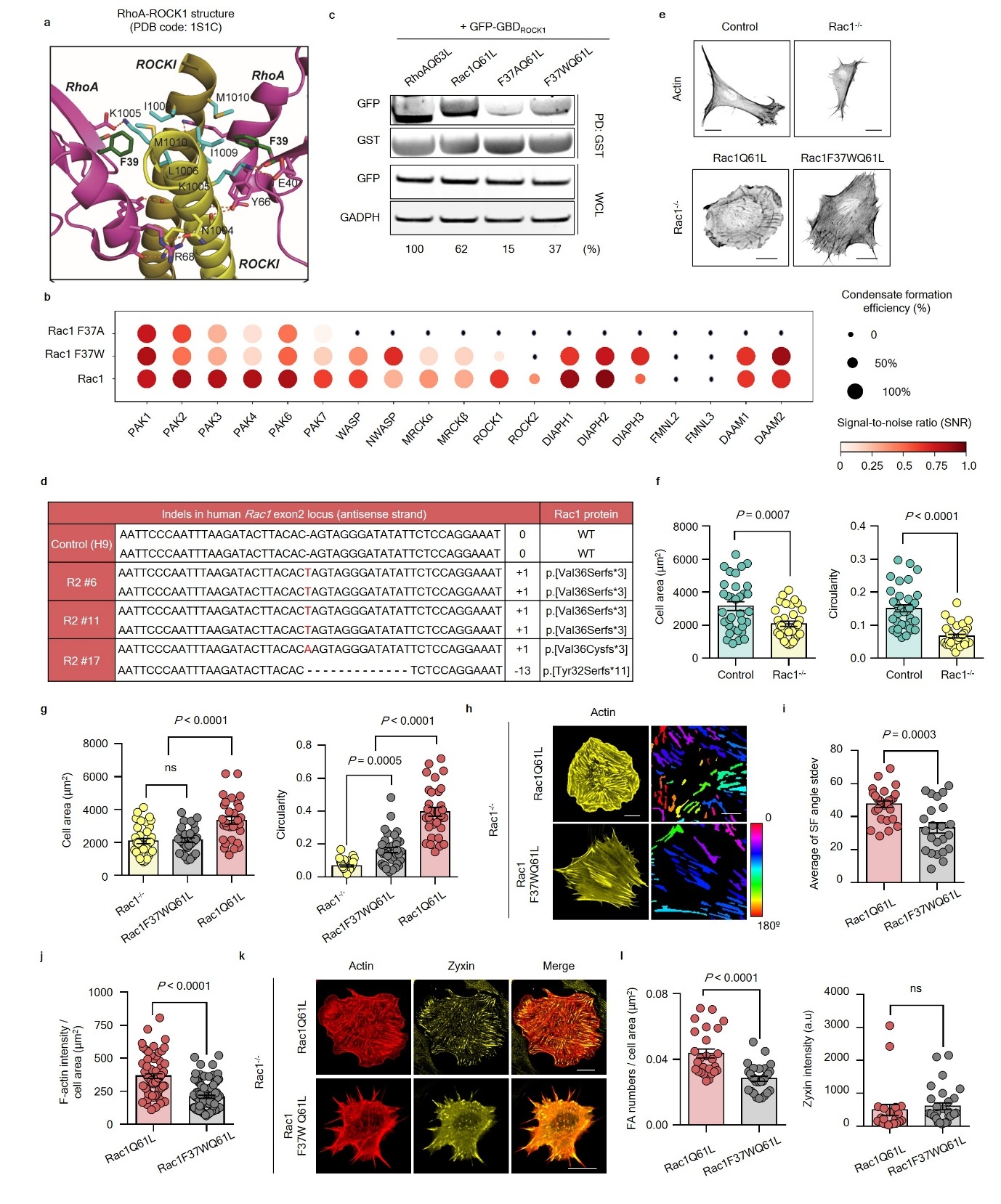
**

**Supplementary Fig. 13. Generation and characterization of Rac/ROCK interaction-impaired mutant (F37W).** (**a**) Crystal structure of RhoA-ROCK1. F39 position of RhoA interacts with the hydrophobic cluster (shown in cyan sticks) consisting of K1005, L1006, I1009, and M1010 of ROCK1. F39W mutation may cause steric hindrance and lead to the disassembly of the complex (PDB code:1S1C). (**b**) Molecular interaction profiles between Rho GTPases and their downstream effectors. Condensate formation efficiency ranges from 0 to 100% and is depicted by the size of the circles. When efficiency is 0, it is displayed in a black dot for better visibility. The signal-to-noise ratio (SNR) ranges from 0-1 and is represented by a one-color gradient within each circle. (**c**) GST pull-down assays validating interactions of GST-RhoAQ63L (DCAAX) or –RacQ61L (DBP and DCAAX) and –RacQ61L mutants (F37A, F37W with DBP and DCAAX) with GFP-GBD_ROCK1_. Western blotting analysis after GST pull-down from whole-cell lysates (WCL) from NIH3T3 cells transfected with GFP-GBD_ROCK1_. The relative signal of each band was normalized to the RhoA (positive control) band, and it is indicated below each band. (**d**) Sequencing results of Control (H9) and Rac1-knockout (Rac1^-/-^) clones. The clones were expanded and differentiated for subsequent experiments. (**e-g**) Representative images and morphological analysis of control (n= 34 cells, N=3) and Rac1^-/-^ (n= 35 cells, N=3) cells expressing mCh-LifeAct (inverted contrast) with CFP-Rac1Q61L or CFP-Rac1F37WQ61L (n= 30 and 32 cells, N=3). Rac1Q61L induced global lamellipodia, but Rac1F37WQ61L failed to induce lamellipodia and showed reduced cell area and circularity. Means and SEM are shown. (**h-j**) Rac1^-/-^ cells co-expressing mCh-LifeAct and CFP-Rac1Q61L or CFP-Rac1F37WQ61L. The degree of actin fibers was calculated and colorized in a range of 0° to 180 °. Graph showing the average stress fiber angle deviation (n= 26 and 23 cells, N=3) and F-actin intensity in Rac1^-/-^ cells expressing CFP-Rac1 or CFP-Rac1F37W (n= 70 and 93 cells, N=3). We observed a significant reduction of F-actin, but the remaining F-actin appeared highly aligned. Means and SEM are shown. (**k, l**) Rac1^-/-^ cells expressing mCh-LifeAct, Zyxin-YFP, and CFP-Rac1Q61L or CFP-Rac1F37WQ61L. Graphs showing focal adhesion numbers per cell area (n= 26 and 29 cells, N=3) and zyxin intensity (n= 21 and 29 cells, N=3). The adhesion numbers decreased in Rac1F37WQ61L compared to Rac1Q61L, but there was no difference in zyxin intensity. Means and SEM are shown. Representative images shown are from ≥ 3 independent experiments with similar results. Scale bars, 20 μm (all panels). *P*-values were determined by the one-way ANOVA with Turkey’s post hoc test in (g). Unpaired two-tailed t-test P-values are shown in (f), (i), (j) and (l).


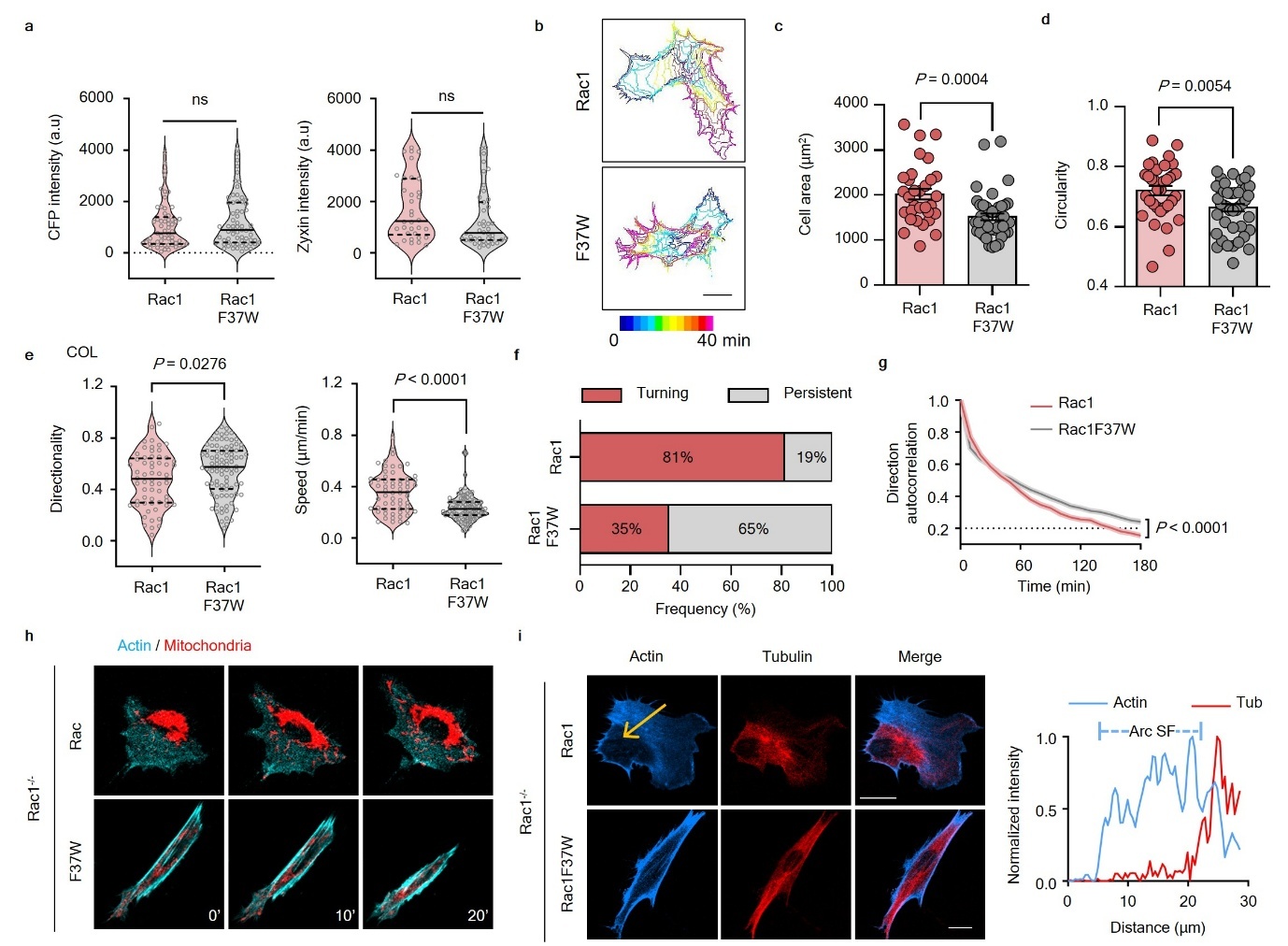


**Supplementary Fig. 14. Rac/ROCK-induced contractility alters cell migration, providing an intrinsic mechanism for orthogonal turning.** (**a**) Graph showing Rac1 and Rac1F37W expression levels (n= 57 and 65 cells, N=3) and zyxin intensity in Rac1^-/-^ cells co-expressing either CFP-Rac1 or CFP-Rac1F37W (n= 34 and 43 cells, N=3). There was no difference in CFP intensity and zyxin intensity. Median value, upper and lower quartiles (25th and 75th percentiles, dotted line) are shown. (**b**) The color-coded outline indicates the migration behavior of Rac1^-/-^ cells expressing either CFP-Rac1 or CFP-Rac1F37W over a period of 40 min (1 min interval). (**c, d**) Quantification of cell area (n=31 and 43 cells, N=3) and circularity in Rac1^-/-^ cells co-expressing either CFP-Rac1 or CFP-Rac1F37W (n=34 and 43 cells, N=3). Means and SEM are shown. (**e**) Analysis of speed and directionality in Rac1^-/-^ cells expressing either CFP-Rac1 (n = 61 cells) or CFP-Rac1F37W (n = 93 cells) and cultured on collagen (5 μg/mL). Median value, upper and lower quartiles (25th and 75th percentiles, dotted line) are shown. (**f**) Quantification of frequencies of turning behavior in migrating Rac1^-/-^ cells expressing either CFP-Rac1 or CFP-Rac1F37W. (**g**) Direction autocorrelation analysis of Rac1^-/-^ cells expressing either CFP-Rac1 (pink, n= 85 cells) or CFP-Rac1F37W (gray, n= 99 cells). A two-way repeated measures ANOVA revealed significant differences between the groups starting at 40 minutes and persisting thereafter (p < 0.0001). Rac1-expressing cells showed a more rapid decrease in autocorrelation compared to Rac1F37W-expressing cells. Means and SEM are shown. (**h**) Representative images of Rac1^-/-^ cells expressing either CFP-Rac1 (upper) or CFP-Rac1F37W (lower) with iRFP682-LifeAct (cyan) and MitoTracker (Red, mitochondria). Images show that mitochondria do not penetrate but accumulate behind the arc SFs. (**i**) Representative images of Rac1^-/-^ cells expressing either CFP-Rac1 or CFP-Rac1F37W with iRFP682-Lifeact (blue) and FuRed-Tubulin (red). Images and graphs show that microtubules (MTs) do not penetrate and instead accumulate behind the arc SFs. Representative images shown are from ≥ 3 independent experiments with similar results. Scale bars, 20 μm (all panels). Unpaired two-tailed t-test P-values are shown in (a), (c), (d) and (e). *P*-values were determined by the two-way ANOVA with Bonferroni’s post hoc test in (g).


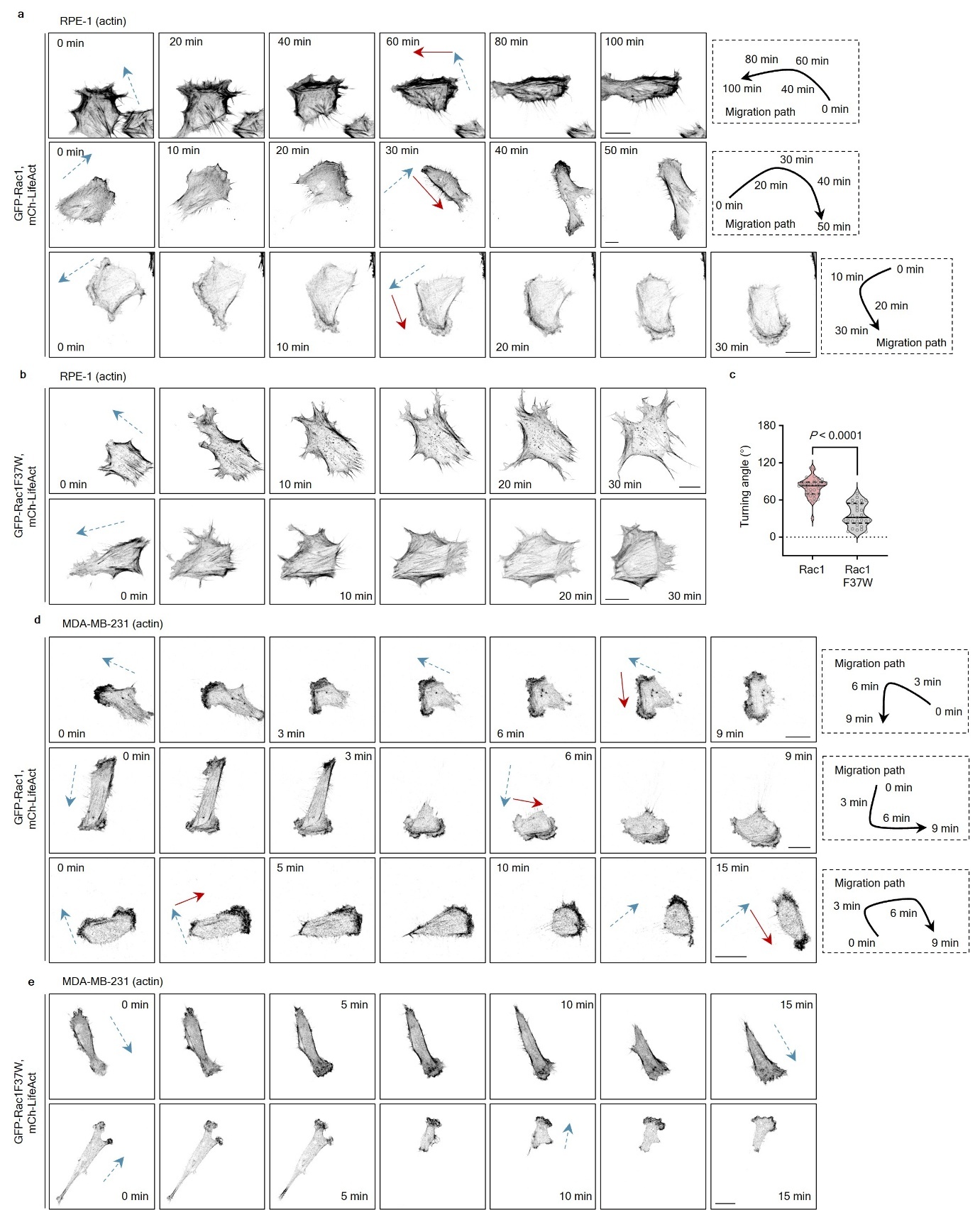


**Supplementary Fig. 15. Rac/ROCK-induced contractility triggering orthogonal turning in different cell types.** (**a**, **b**) Time-lapse images of mCh-LifeAct (inverted contrast) in migrating RPE-1 cells expressing GFP-Rac1 (a) and GFP-Rac1F37W (b). The blue arrows indicate the direction of the cell moving, where the tail retraction occurs, while the red arrows point to the direction of newly formed protrusions following tail retraction. (**c**) Graph showing the angle between protrusions formed after tail retraction in migrating RPE-1 cells expressing GFP-Rac1 and GFP-Rac1F37W (n= 35 and 28 cells, N=3). Median value, upper and lower quartiles (25th and 75th percentiles, dotted line) are shown. (**d**, **e**) Time-lapse images of mCh-LifeAct (inverted contrast) in migrating MDA-MB-231 cells expressing GFP-Rac1 (c) and GFP-Rac1F37W (d). In both RPE-1 and MDA-MB-231, cells expressing Rac1F37W showed impaired arc SF formation and reduced turning. Representative images shown are from ≥ 3 independent experiments with similar results. Scale bars, 20 μm (all panels). Unpaired two-tailed t-test P-values is shown in (c).

**
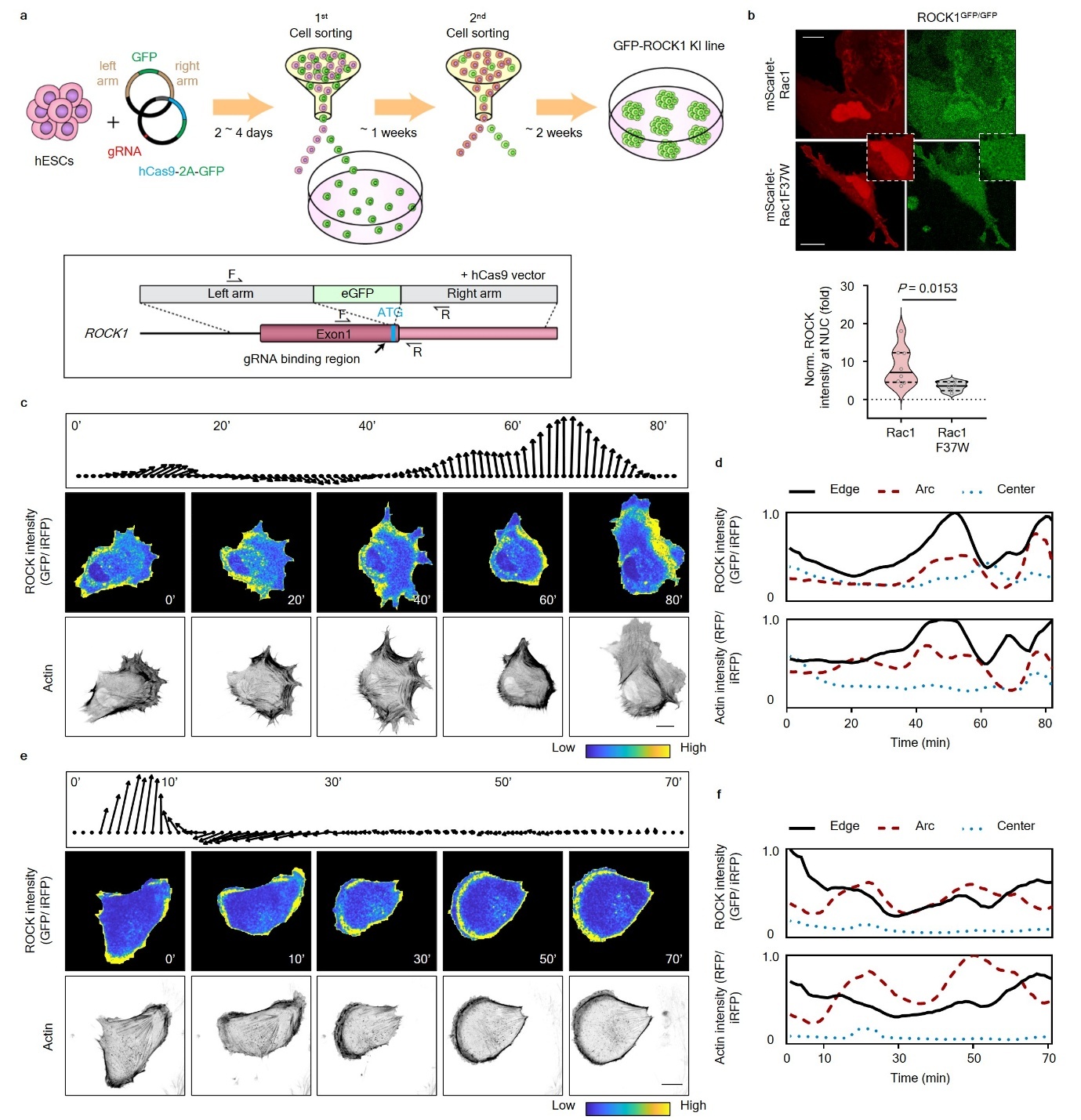
**

**Supplementary Fig. 16 Observation and analysis of ROCK and Rac1 localization through GFP knockin using the CRISPR/Cas9 system.** (**a**) Diagram illustrating the generation of hESC lines with GFP knockin in the ROCK1 locus using the CRISPR/Cas9 system. Schematic diagram of ROCK1 gene targeting using homologous recombination enhanced by CRISPR/Cas9 system. (**b**) Representative images of ROCK1^GFP/GFP^ cells expressing mScarlet-Rac1 or mScarlet-Rac1F37W. ROCK shows strong nuclear localization when Rac1 strongly localizes the nucleus, while ROCK is not localized in the nucleus in adjacent cells where Rac1 is not expressed or absent from the nucleus. Cells expressing mScarlet-Rac1F37W (n= 8 cells, N=3) also showed strong nuclear localization and increased ROCK nuclear localization, but a significant difference in nuclear ROCK intensity was observed compared to Rac1-expressing cells (n= 6 cells, N=3). Median value, upper and lower quartiles (25th and 75th percentiles, dotted line) are shown. (**c-f**) Representative time-series profiles for the normalized ROCK and Actin intensities and cell migration behaviors. The notations are identical to Fig. 5d and e. Representative images shown are from ≥ 3 independent experiments with similar results. Scale bars, 20 μm (all panels). Unpaired two-tailed t-test P-values is shown in (b).


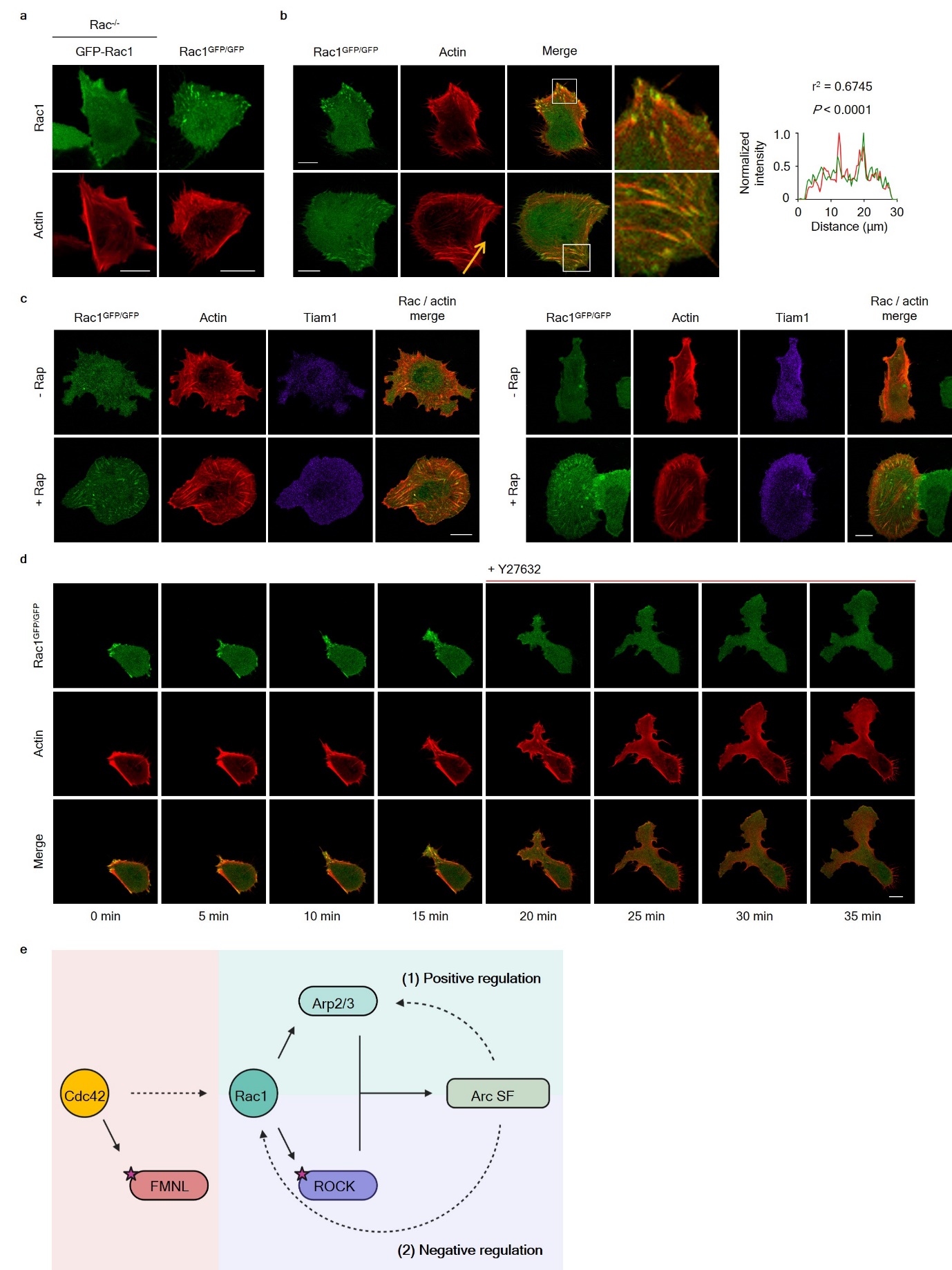


**Supplementary Fig. 17. Rac1/ROCK/MLC-mediated contractility facilitates the localization of Rac1 at adhesions, preventing the formation of excessive protrusions.** (**a, b**) Rac1 localization was compared between Rac1^-/-^ cells expressing GFP-Rac1 with mCh-LifeAct and Rac1^GFP/GFP^ cells expressing mCh-LifeAct. Cells were cultured on 1 μg/mL FN. Endogenous GFP-tagged Rac1 showed a clear localization pattern. Rac1 prominently co-localized at the termini of actin filaments. Graph showing a correlation between Rac1 and actin intensity and the correlation coefficient (r^2^) was shown. (**c**) Rac1^GFP/GFP^ cells expressing Lyn-FRB, iRFP-FKBP-Tiam1, mCh-LifeAct. Cells were treated with 500 nM rapamycin. Images were captured 1 hour after rapamycin treatment. (**d**) Time-lapse images of Rac1^GFP/GFP^ cells expressing mCh-LifeAct. Cells were treated with 20 μM Y27632. (**e**) Schematic illustration shows the intrinsic molecular machinery that facilitates the process from establishing polarity to directional changes regulated by an ensemble of Rho GTPases and effectors. Rac/ROCK/MLC-mediated contractility provides two distinct regulations that increase cellular randomness. The positive regulation (1) is an intrinsic characteristic, while extracellular matrix-dependent adhesion formation influences the negative regulation (2). Ultimately, (1) and (2) prevent persistent cell movement. Representative images shown are from ≥ 3 independent experiments with similar results. Scale bars, 20 μm (all panels). Schematic was created in BioRender. Heo, W. (2025) https://BioRender.com/2718swf.

**Full blot for Supplementary Figure 8b and 13c**

Full blot images corresponding to Supplementary Fig. 8b and 13c are provided in this document.

**
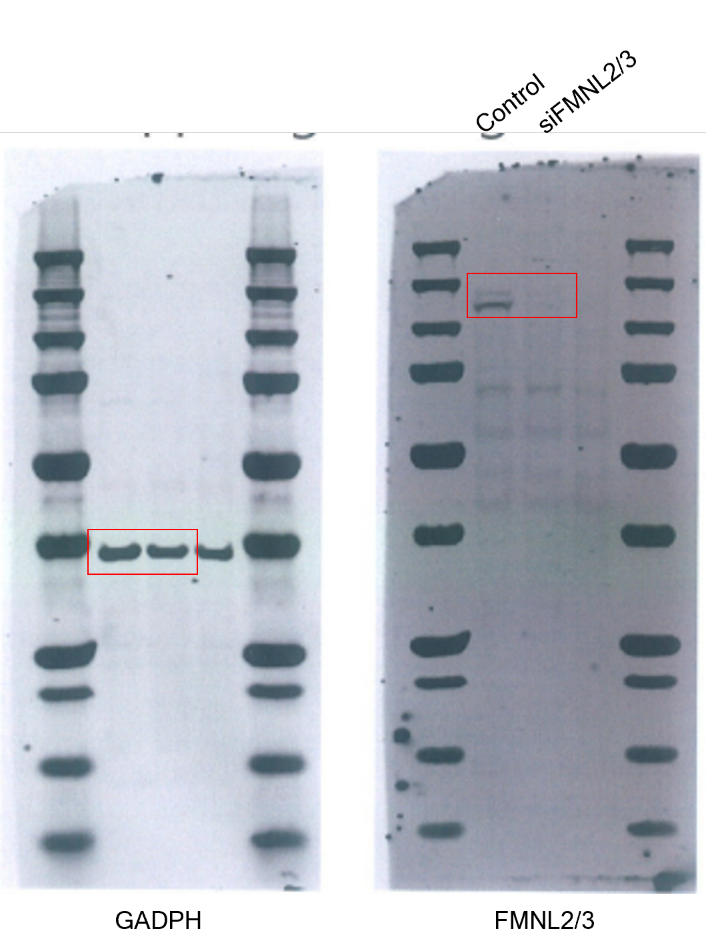
**FMNL2 (cross-reactive to the FMNL3; 1:500, Abcam, ab57963), rabbit anti-GAPDH (1:500, Santa Cruz), goat anti-rabbit IRDye 680RD (1: 10,000; LI-COR), and goat anti-mouse IRDye 800CW (1: 10,000; LI-COR, 926-322210).


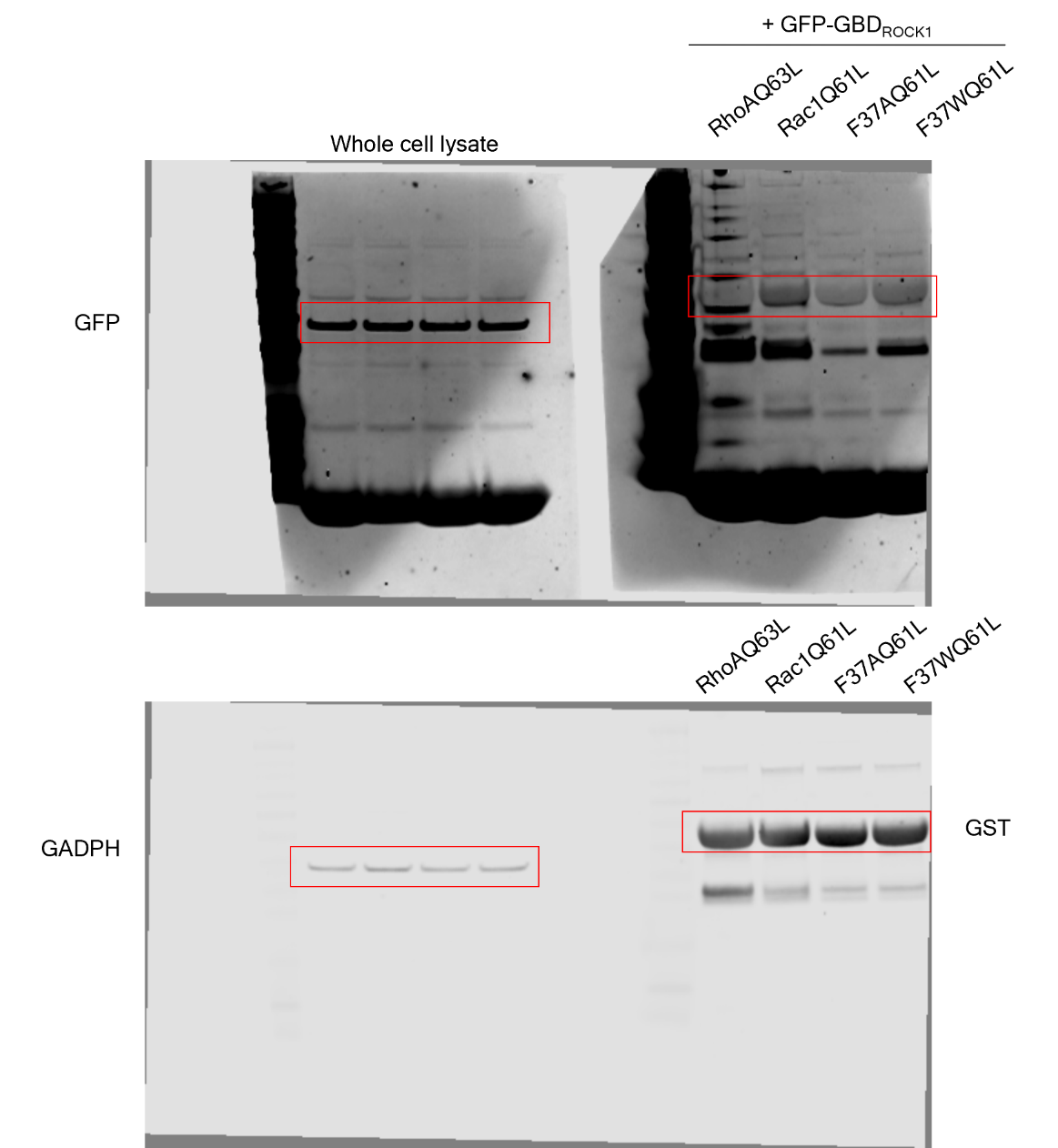
Rabbit anti-GFP (1:300, Santa Cruz, sc-8334), mouse anti-GST (1:300, Santa Cruz, sc-138), and rabbit anti-GAPDH (1:500, Santa Cruz), goat anti-rabbit IRDye 680RD (1: 10,000; LI-COR), and goat anti-mouse IRDye 800CW (1: 10,000; LI-COR, 926-322210).
