# Supplementary material for: A Rho GTPase-effector ensemble governs cell migration behavior
Source: Nat Commun. 2025 Oct 31;16:9637. doi: 10.1038/s41467-025-64635-0 (PMC12579213; doi:10.1038/s41467-025-64635-0)
Supplement: Supplementary file 2 — Description of Additional Supplementary Information [file 41467_2025_64635_MOESM2_ESM.docx]

Supplementary Data. 1. Nucleotide sequences of oligonucleotides used in this study (PCR primers and CRISPR guide RNAs).
