# Supplementary material for: A Rho GTPase-effector ensemble governs cell migration behavior
Source: Nat Commun. 2025 Oct 31;16:9637. doi: 10.1038/s41467-025-64635-0 (PMC12579213; doi:10.1038/s41467-025-64635-0)
Supplement: Supplementary file 4 — Reporting Summary [file 41467_2025_64635_MOESM4_ESM.pdf]

nature portfolio

Corresponding author(s): Gabsang Lee, Kwang-Hyun Cho, Won Do Heo

Last updated by author(s): Sep 12, 2025

Reporting Summary

Nature Portfolio wishes to improve the reproducibility of the work that we publish. This form provides structure for consistency and transparency in reporting. For further information on Nature Portfolio policies, see our Editorial Policies and the Editorial Policy Checklist.

Statistics

For all statistical analyses, confirm that the following items are present in the figure legend, table legend, main text, or Methods section.

n/a	Confirmed	
☐	☒	The exact sample size (*n*) for each experimental group/condition, given as a discrete number and unit of measurement
☐	☒	A statement on whether measurements were taken from distinct samples or whether the same sample was measured repeatedly
☐	☒	The statistical test(s) used AND whether they are one- or two-sided<br>*Only common tests should be described solely by name; describe more complex techniques in the Methods section.*
☒	☐	A description of all covariates tested
☐	☒	A description of any assumptions or corrections, such as tests of normality and adjustment for multiple comparisons
☐	☒	A full description of the statistical parameters including central tendency (e.g. means) or other basic estimates (e.g. regression coefficient) AND variation (e.g. standard deviation) or associated estimates of uncertainty (e.g. confidence intervals)
☐	☒	For null hypothesis testing, the test statistic (e.g. *F*, *t*, *r*) with confidence intervals, effect sizes, degrees of freedom and *P* value noted<br>*Give P values as exact values whenever suitable.*
☒	☐	For Bayesian analysis, information on the choice of priors and Markov chain Monte Carlo settings
☒	☐	For hierarchical and complex designs, identification of the appropriate level for tests and full reporting of outcomes
☐	☒	Estimates of effect sizes (e.g. Cohen's *d*, Pearson's *r*), indicating how they were calculated

Our web collection on statistics for biologists contains articles on many of the points above.

Software and code

Policy information about availability of computer code

Data collection	Nikon AIR confocal microscope mounted on a Nikon Eclipse Ti body with a Nikon CFI Plan Apochromat 60X/l.4 numerical aperture (NA) and NIS-elements AR 64-bit version 5.21 are used for fluorescence image.
Data analysis	Data was analyzed in Graphpad Prism 10 and Microsoft Excel. Imaging data was analyzed in Nikon imaging software 5.2 1. was used for confocal images.

For manuscripts utilizing custom algorithms or software that are central to the research but not yet described in published literature, software must be made available to editors and reviewers. We strongly encourage code deposition in a community repository (e.g. GitHub). See the Nature Portfolio guidelines for submitting code & software for further information.

Data

Policy information about availability of data

All manuscripts must include a data availability statement. This statement should provide the following information, where applicable:

- Accession codes, unique identifiers, or web links for publicly available datasets
- A description of any restrictions on data availability
- For clinical datasets or third party data, please ensure that the statement adheres to our policy

We have provided a full data availability statement in the manuscript. All quantified data are included in the Source Data files. Due to file size limitations, the raw confocal images have not been uploaded but are available from the corresponding author upon reasonable request.

Research involving human participants, their data, or biological material

Policy information about studies with human participants or human data. See also policy information about sex, gender (identity/presentation), and sexual orientation and race, ethnicity and racism.

Reporting on sex and gender	N/A
Reporting on race, ethnicity, or other socially relevant groupings	N/A
Population characteristics	N/A
Recruitment	N/A
Ethics oversight	N/A

Note that full information on the approval of the study protocol must also be provided in the manuscript.

Field-specific reporting

Please select the one below that is the best fit for your research. If you are not sure, read the appropriate sections before making your selection.

[x] Life sciences   [ ] Behavioural & social sciences   [ ] Ecological, evolutionary & environmental sciences

For a reference copy of the document with all sections, see nature.com/documents/nr-reporting-summary-flat.pdf

Life sciences study design

All studies must disclose on these points even when the disclosure is negative.

Sample size	All experiments were performed with at least three independent biological replicates.
Data exclusions	No data was excluded.
Replication	All experiments at replications were successful.
Randomization	Cells were randomly assigned to experimental groups.
Blinding	This is not relevant to cell biology studies and samples were not blinded.

Reporting for specific materials, systems and methods

We require information from authors about some types of materials, experimental systems and methods used in many studies. Here, indicate whether each material, system or method listed is relevant to your study. If you are not sure if a list item applies to your research, read the appropriate section before selecting a response.

Materials & experimental systems

n/a	Involved in the study	
[ ]	[x]	Antibodies
[ ]	[x]	Eukaryotic cell lines
[x]	[ ]	Palaeontology and archaeology
[x]	[ ]	Animals and other organisms
[x]	[ ]	Clinical data
[x]	[ ]	Dual use research of concern
[x]	[ ]	Plants

Methods

n/a	Involved in the study	
[x]	[ ]	ChIP-seq
[x]	[ ]	Flow cytometry
[x]	[ ]	MRI-based neuroimaging

Antibodies

Antibodies used	Rabbit anti-ROCKl monoclonal anti body (sc-5560, Santacruz), anti-ROCK2 (Santacruz, sc-1851) and phosphor-MLC2 (Cell signal ing, 3671), antibodies: rabbit anti-GAPDH{Santacruz, sc-51907), rabbit anti-phosphor-MLC2 (Cell signaling, 3671), rabbit anti-GFP (Santacruz, sc-8334), mouse anti-FMNL2 (cross-reactive to the FMNL3; 1:500, Abcam, ab57963), mouse anti-GST (Santacruz, sc-138), Alexa Fluor 647-conjugated goat anti -rabbit lgG (Thermo Fisher Scientific, A-21244), goat anti-rabbit IRDye 680RD (LI-COR, 926-68071), goat anti-mouse IRDye 800CW (LI-COR, 926-322210)
Validation	Validation Antibodies were only chosen if there were validated references available.

Eukaryotic cell lines

Policy information about cell lines and Sex and Gender in Research

Cell line source(s)	Undifferentiated H9 (WA09, WiCell), mouse embryonic fi broblasts (MEFs; Applied Stem Cell, ASF-1213). HeLa (CCL-2), A549 (CCL-185), MDAMB-231 (HTB-26), HT1080 (CCL-121) and HK-2 (CRL-2190), hTERT-immortalized retinal pigment epithelial (CRL-4000), U-20S (HTB-96) and NIH 3T3 celll ines were acquired from ATCC.
Authentication	None of the celll ine used were authenticated.
Mycoplasma contamination	The cell line tested negative for mycoplasma contamination.
Commonly misidentified lines (See ICLAC register)	No commonly misidentified cell line were used.

Plants

Seed stocks	N/A
Novel plant genotypes	N/A
Authentication	N/A
